# Supplementary material for: A propolis-derived small molecule ameliorates metabolic syndrome in obese mice by targeting the CREB/CRTC2 transcriptional complex
Source: Nat Commun. 2022 Jan 11;13:246. doi: 10.1038/s41467-021-27533-9 (PMC8752738; doi:10.1038/s41467-021-27533-9)
Supplement: Supplementary file 1 — Supplementary Information [file 41467_2021_27533_MOESM1_ESM.pdf]

Supplementary Materials

A Propolis-Derived Small Molecule Ameliorates Metabolic Syndrome in Obese Mice by Targeting the CREB/CRTC2 Transcriptional Complex

Yaqiong Chen[1,8], Jiang Wang[2,8], Yibing Wang[2,3], Pengfei Wang[3,4], Zan Zhou[3,4], Rong Wu[3,4], Qian Xu[5], Hanyun You[6], Yaxin Liu[1], Lei Wang[1], Lingqin Zhou[1], Yuting Wu[4], Lihong Hu[6,9]✉, Hong Liu[2,9]✉& Yi Liu [1,7,9]✉

[1] Key Laboratory of Metabolism and Molecular Medicine, the Ministry of Education, Department of Biochemistry and Molecular Biology, School of Basic Medical Sciences, Fudan University, 200032 Shanghai, China.
[2] State Key Laboratory of Drug Research and CAS Key Laboratory of Receptor Research, Shanghai Institute of Materia Medica, Chinese Academy of Sciences, 201203 Shanghai, China.
[3] University of Chinese Academy of Sciences, No.19A Yuquan Road, 100049 Beijing, China.
[4] Key Laboratory of Nutrition and Metabolism, Institute for Nutritional Sciences, Shanghai Institutes for Biological Sciences, 200031 Shanghai, China.
[5] Department of Endocrinology, the First Affiliated Hospital of Harbin Medical University, 150081 Heilongjiang Province, China.
[6] Jiangsu Key Laboratory for Functional Substance of Chinese Medicine, School of Pharmacy, Nanjing University of Chinese Medicine, 210023 Nanjing, China.
[7] State Key Laboratory of Medical Neurobiology and MOE Frontiers Center for Brain Science, School of Basic Medical Sciences, Fudan University, 200032 Shanghai, China.
[8] These authors contributed equally: Yaqiong Chen, Jiang Wang.
[9] These authors jointly supervised this work: Lihong Hu, Hong Liu, Yi Liu.
✉email: lhhu@njucm.edu.cn; hliu@simm.ac.cn; liuyee@fudan.edu.cn

Supplementary Fig. 1

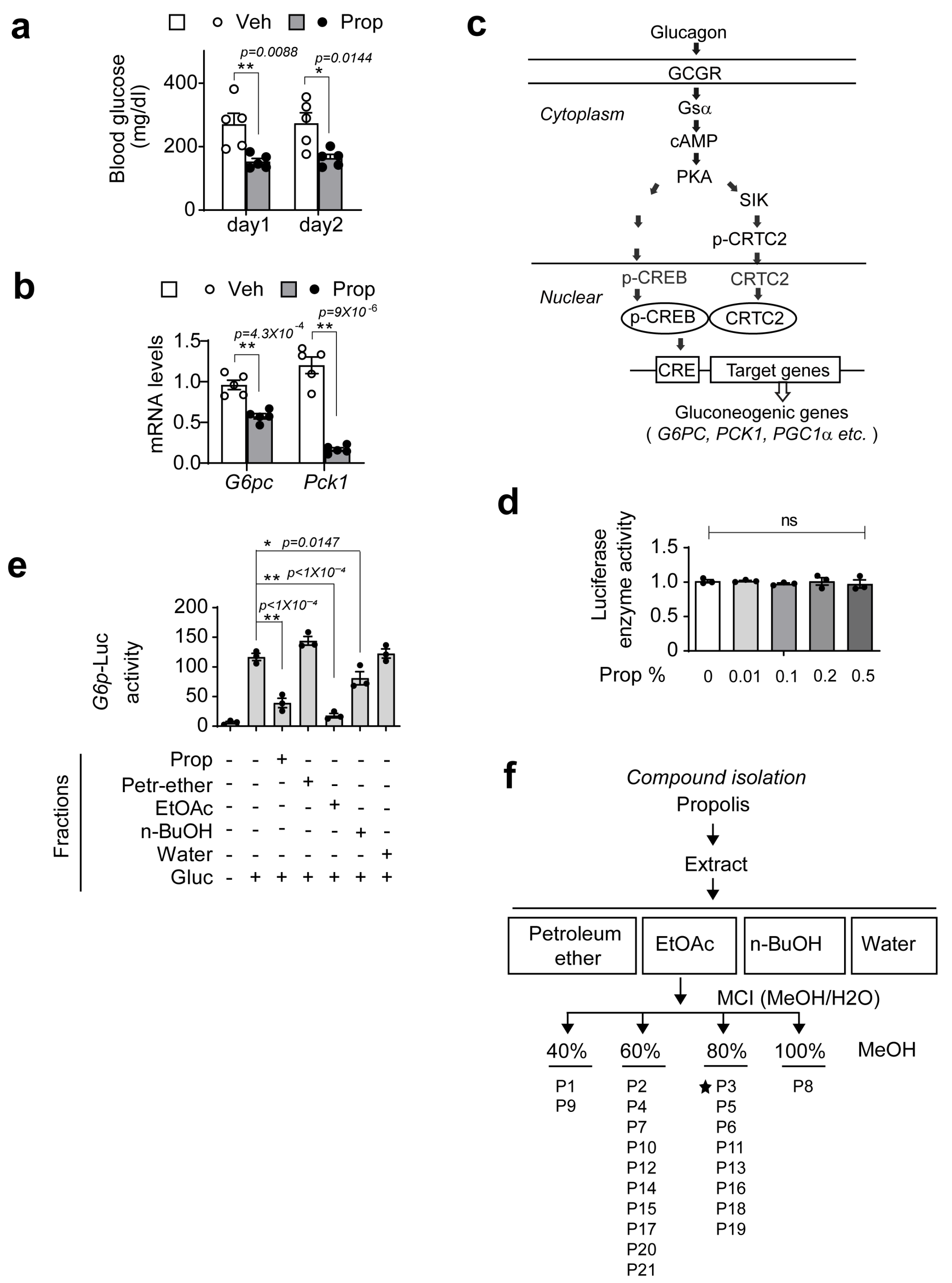

Supplementary Figure 1. Identifying inhibitors of gluconeogenesis from Brazilian green propolis

a) Measurement of blood glucose levels in 16-h-fasted *db/db* mice administered Brazilian green propolis (250 mg/kg) or vehicle orally one time daily for 2 days. One of three independent experiments presented here. Data are represented as mean ± SEM (n=5 per group). *, $p < 0.05$; **, $p < 0.01$; p values were determined by two-way ANOVA followed Bonferroni's multiple comparisons test.

b) Quantitative PCR analysis of hepatic gluconeogenic gene expression in *db/db* mice administered propolis (250 mg/kg) or vehicle one time daily orally for 3 weeks. One of two independent experiments presented here. Data are represented as mean ± SEM (n=5 per group). *, $p < 0.05$; **, $p < 0.01$; p values were determined by unpaired two-tailed multiple t test with two-stage linear step-up procedure, each gene was analyzed individually, without assuming a consistent SD.

c) Diagram of CREB/CRTC2-mediated gluconeogenic transcription induced by glucagon.

d) Luciferase enzyme activity. The luciferase enzymatic activity from HEK293T cells incubated with propolis. The cells ectopically expressed luciferase (*CRE-Luc*) and were stimulated with FSK (10 nM) for 5 h before lysis, and then incubated with various concentrations of propolis for 30 min before luciferase activity determination. One of two independent experiments presented here. Data are represented as mean ± SEM (n=3 per treatment). ns, $p > 0.05$; *, $p < 0.05$; **, $p < 0.01$; p values were determined by one-way ANOVA followed Dunnett's multiple comparisons test.

e) Measure the bio-activity from propolis fractions. The *G6P-Luc* activity in primary hepatocytes treated with propolis (Prop) or indicated propolis fractions for 1-h prior to 6-h stimulation with glucagon (Gluc, 100 nM). The contents of propolis fractions were equal to that in 0.2% propolis. One

of two independent experiments presented here. Data are represented as mean ± SEM (n=3 per treatment). ns, $p > 0.05$; *, $p < 0.05$; **, $p < 0.01$; p values were determined by one-way ANOVA followed Dunnett's multiple comparisons test.

f) Flowchart of compound isolation from Brazilian green propolis. P3 indicated by star in compound list was derived from the EtOAc fraction. Source data for this figure are provided as a Source data file.

Supplementary Fig. 2

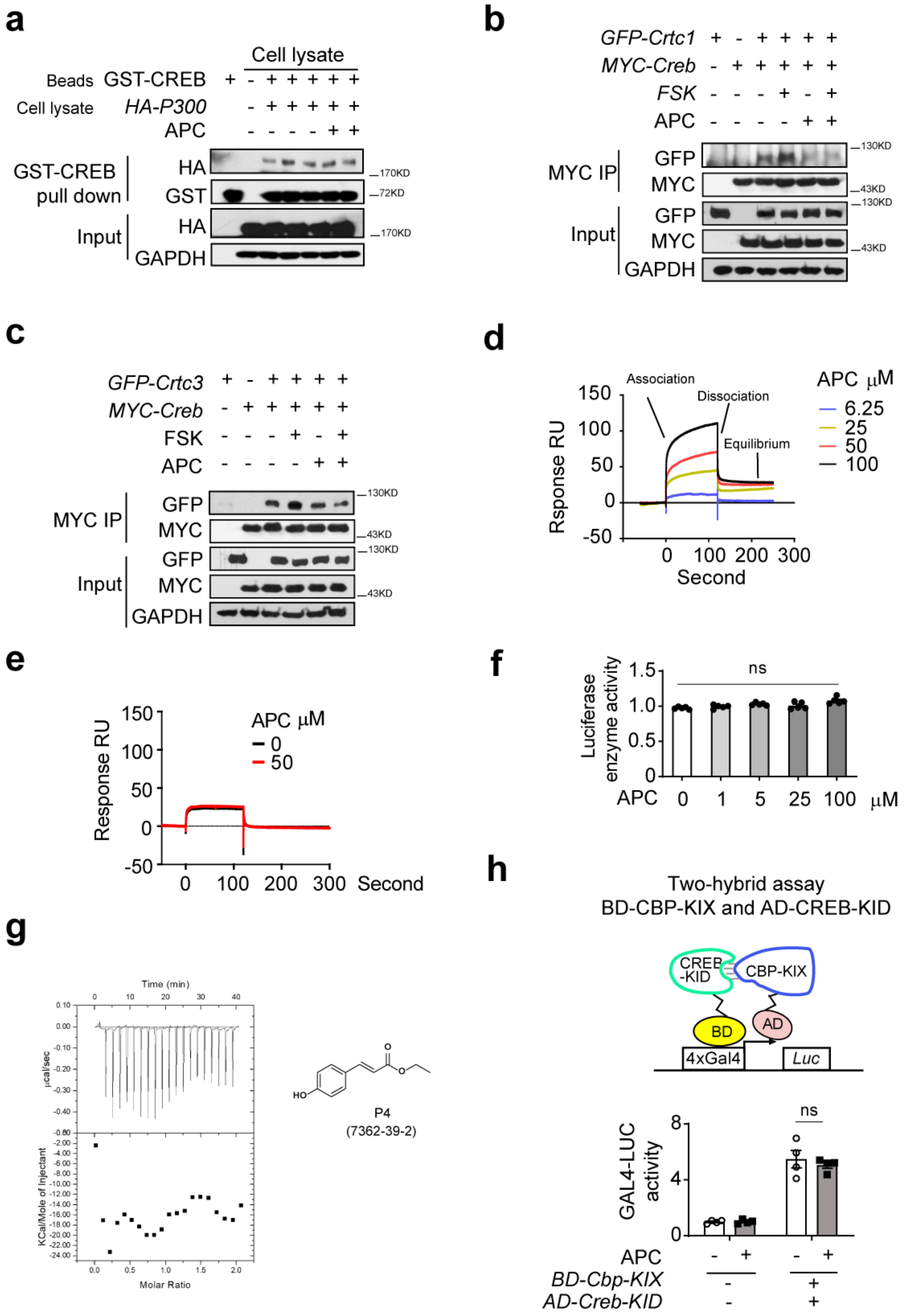

Supplementary Figure 2. APC binds with CREB but does not affect CREB-P300 interaction

a) Immunoblot of HA-P300 recovered from HEK293T cell lysate using GST-CREB beads. APC (10 μM) was incubated with cells for 1-h before FKS (10 nM) stimulation and was included in the following pull-down mixtures (*n*=3). One of two independent experiments presented here.

b) Immunoblot for recovered GFP-CRTC1 or **(c)** GFP-CRTC3 immunoprecipitated by MYC-CREB from overexpression HEK293T cell line. APC (10 μM) was incubated with cells for 1-h before FKS (10 nM) stimulation and was included in the following pull-down mixtures. One of two independent experiments presented here.

d) SPR sonogram traces of APC binding with HIS-CREB proteins immobilized to NTA-chip. The concentration series of APC was as indicated.

e) SPR sonogram traces of APC flowing through HIS-CRTC2-S171A proteins immobilized on NTA-chip. The concentration of APC was as indicated.

f) The luciferase enzymatic activity of HEK293T incubated with APC. Cells over-expressed luciferase CRE-Luc reporter and were stimulated by FSK (10 nM) for 5-h before lysis, which was then incubated with APC (concentration indicated) for 30 min before luciferase activity determination (*n*=3 per treatment). Data are represented as mean ± SEM. *, $p < 0.05$; **, $p < 0.01$; *p* values were determined by one-way ANOVA followed Dunnett's multiple comparisons test.

g) Isothermal titration calorimetry (ITC) of HIS-CREB protein solution titrated into compound P4, *p*-Coumaric acid ethyl ester, solution (left).

h) Reporter activity by two-hybrid assay based on the protein interaction between BD-CREB-KID and AD-CBP-KIX in HEK293T cells incubated with APC (10 μM) for 8-h. A schematic diagram of

this assay is shown at right. Data are represented as mean ± SEM. (n=4 per treatment). *, $p < 0.05$; **, $p < 0.01$; p values were determined by two-way ANOVA repeated measures followed Bonferroni test. Source data for this figure are provided as a Source data file.

Supplementary Fig. 3

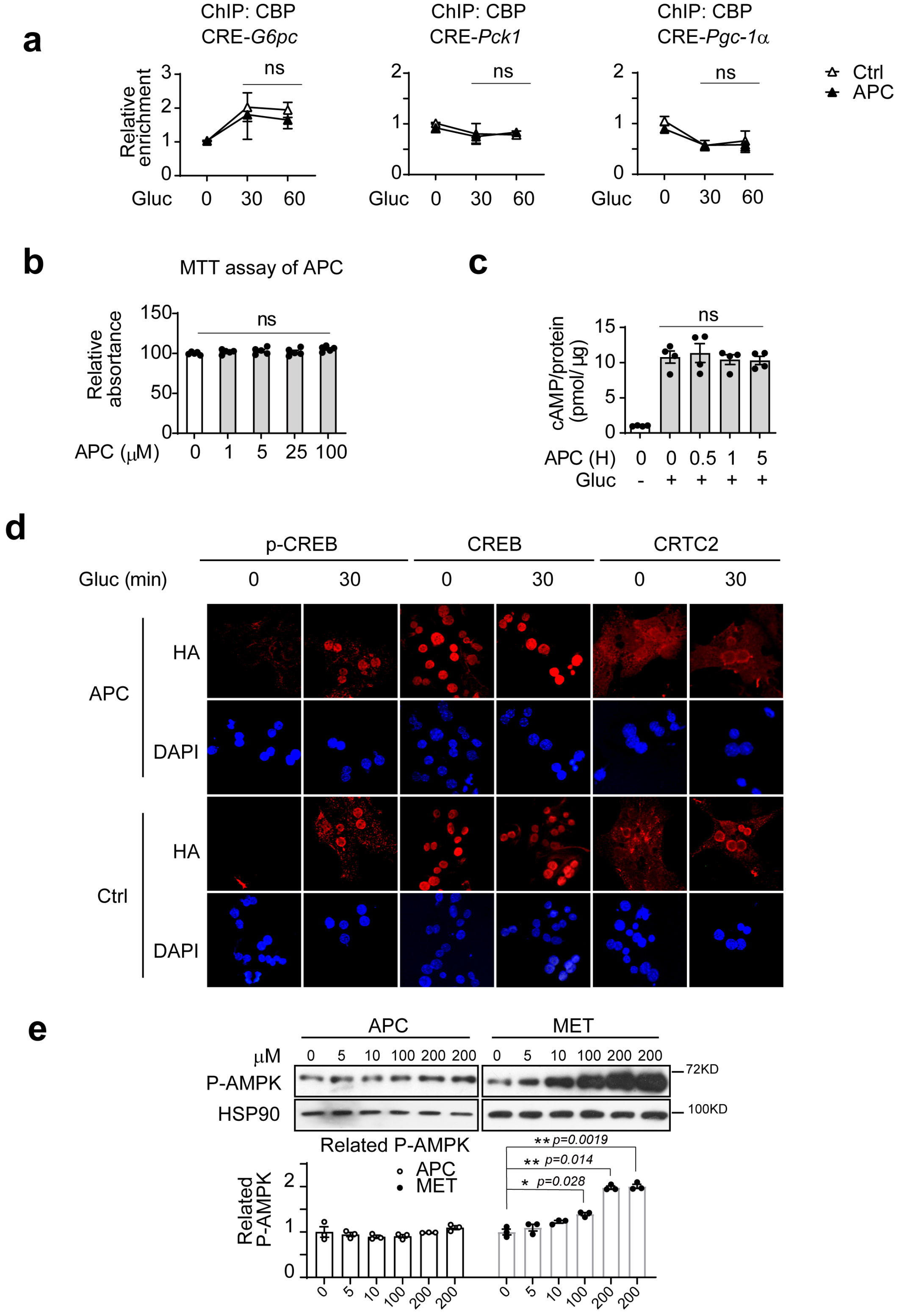

1

2

Supplementary Figure 3. APC inhibits gluconeogenesis independent of CREB-phosphorylation, cell toxin or AMPK activation.

a) CBP occupancy over the CRE sites in the promoters of *G6pc*, *Pck1* and *Pgc-1*α in primary mouse hepatocytes overexpressing HA-CRTC2. Pretreatment with APC (10 μM) for 1-h prior to stimulation with glucagon (Gluc, 100 nM) for indicated time (0, 30, 60 min). One of three independent experiments is presented here. Data are represented as mean ± SEM (n=4 per treatment). ns, p>0.05; *, $p < 0.05$; **, $p < 0.01$; p values were determined by two-way ANOVA followed Bonferroni's multiple comparisons test.

b) MTT assay with primary hepatocytes exposed to APC (1–100 μM) for 48-h (n=5 per treatment). Data are represented as mean ± SEM. ns, p>0.05; *, $p < 0.05$; **, $p < 0.01$; p values were determined by one-way ANOVA followed Dunnett's multiple comparisons test.

c) The cAMP levels of primary hepatocytes exposed to APC (10 μM) for indicated times followed by 30 min stimulation with glucagon (Gluc, 100 nM). The relative cAMP levels were normalized by total protein concentrations determined by BCA assay (n=4 per treatment). One of three independent experiments is presented here. ns, p>0.05; *, $p < 0.05$; **, $p < 0.01$; p values were determined by one-way ANOVA followed Dunnett's multiple comparisons test.

d) Immunostaining of Ser133-phosphorylated CREB, CREB and HA-CRTC2 in primary hepatocytes exposed to APC (10 μM) for 1-h prior to 30 min stimulation with glucagon (100 nM). Nuclei were stained with DAPI. One of three independent experiments is presented here.

e) Phosphate-AMPK level in primary hepatocytes incubated with APC or metformin (MET) at indicated concentrations (0–200 μM) for 30 minutes (top). Relative P-AMPK normalized by AMPK is

presented as bar graph (bottom, $n=3$). One of three independent experiments is presented here. ns, $p>0.05$; *, $p < 0.05$; **, $p < 0.01$; p values were determined by two-way ANOVA followed Bonferroni's multiple comparisons test. Source data for this figure are provided as a Source data file.

Supplementary Fig. 4

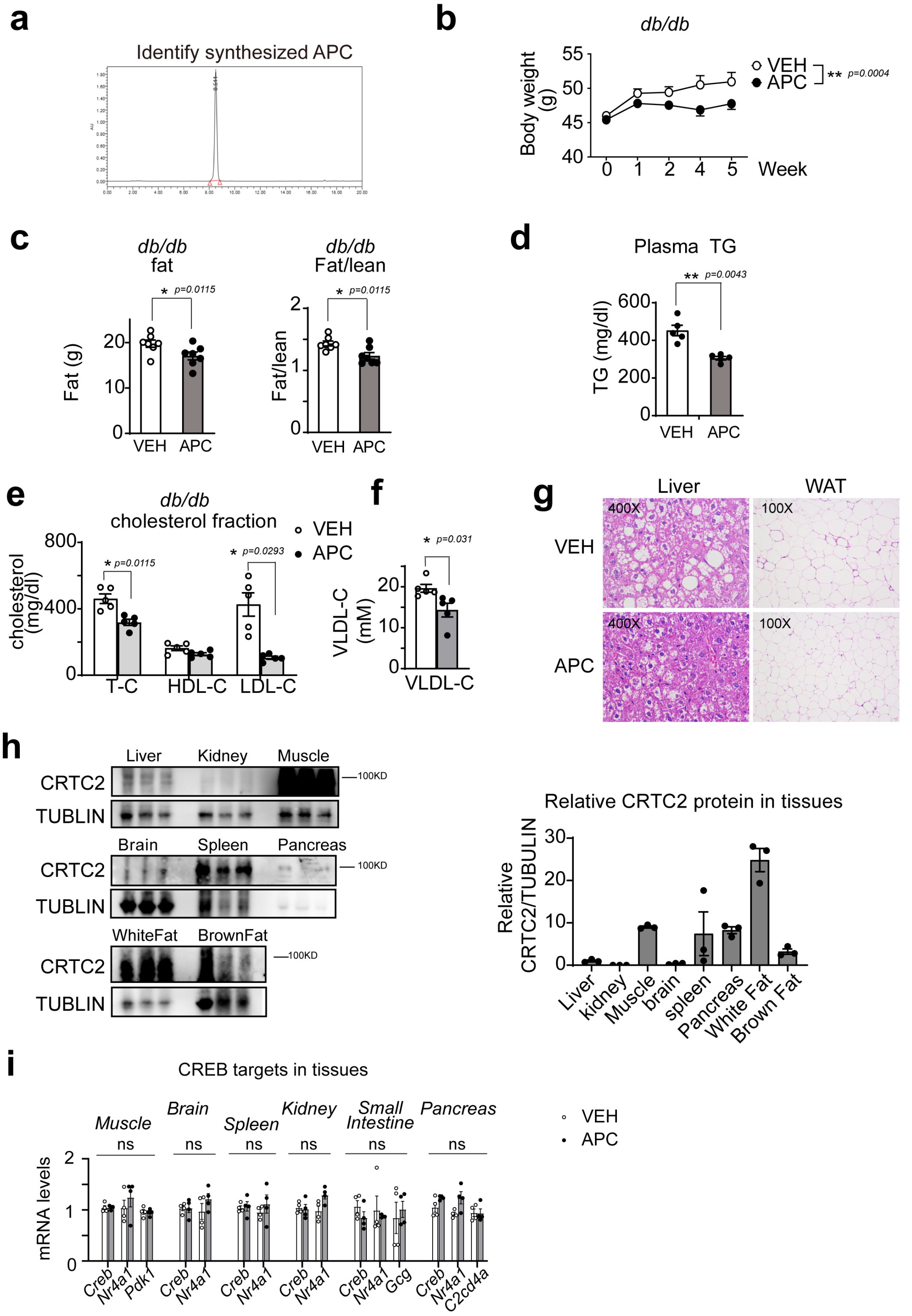

Supplementary Figure 4. APC improves hypolipidemia in *db/db* mice *in vivo*

db/db mice were continuously *i.p.* injected with either synthesized APC at indicated doses (APC 20 mg/kg) or vehicle (VEH) one time daily for 5 weeks (n = 5–7).

a) HPLC identification of synthesized APC.

b) Body weight of these mice. Data are represented as mean ± SEM (n=6 per group). *, $p < 0.05$; **, $p < 0.01$; p values were determined by two-way ANOVA followed Bonferroni's multiple comparisons test.

c) Fat mass (left) and ratio of fat to lean (right) in treated *db/db* mice. Data are represented as mean ± SEM (n=7 per group). *, $P < 0.05$; **, $p < 0.01$, p values were determined by unpaired two-tailed t test with Welch's correction.

d) Plasma TG in treated *db/db* mice. Data are represented as mean ± SEM (n=5 per group). *, $P < 0.05$; **, $p < 0.01$, p values were determined by unpaired two-tailed t test with Welch's test.

e) The content of serum total cholesterol (TC), LDL cholesterol (LDL-C), HDL cholesterol (HDL-C) and (**f**) VLDL cholesterol (VLDL-C) in treated *db/db* mice. Data are represented as mean ± SEM (n=5 per group). *, $p < 0.05$; **, $p < 0.01$; p values were determined by two-way ANOVA followed Bonferroni's multiple comparisons test.

g) H&E stain of liver and white adipose tissue in treated *db/db* mice. Data are represented as mean ± SEM (n=5 per group). *, $P < 0.05$; **, $p < 0.01$, p values were determined by unpaired two-tailed t test with Welch's test.

h) Immunoblotting of endogenic CRTC2 in tissues from wild type C57BL6 mice (left), and relative CRTC2 levels normalized by TUBULIN are shown as bar graph (right).

i) The mRNA levels of CREB target genes in different tissues of male wild C57BL6 mice, which oral administrated one dose of APC (20 mg/kg) or vehicle after fasting 12-h and for another 6-h fasting before being sacrificed. Data are represented as mean ± SEM (n=4 per group). ns, p>0.05; *, $P < 0.05$; **, $p < 0.01$; p values were determined by unpaired two-tailed multiple t test with two-stage linear step-up procedure, each gene was analyzed individually, without assuming a consistent SD. Source data for this figure are provided as a Source data file.

Supplementary Fig. 5

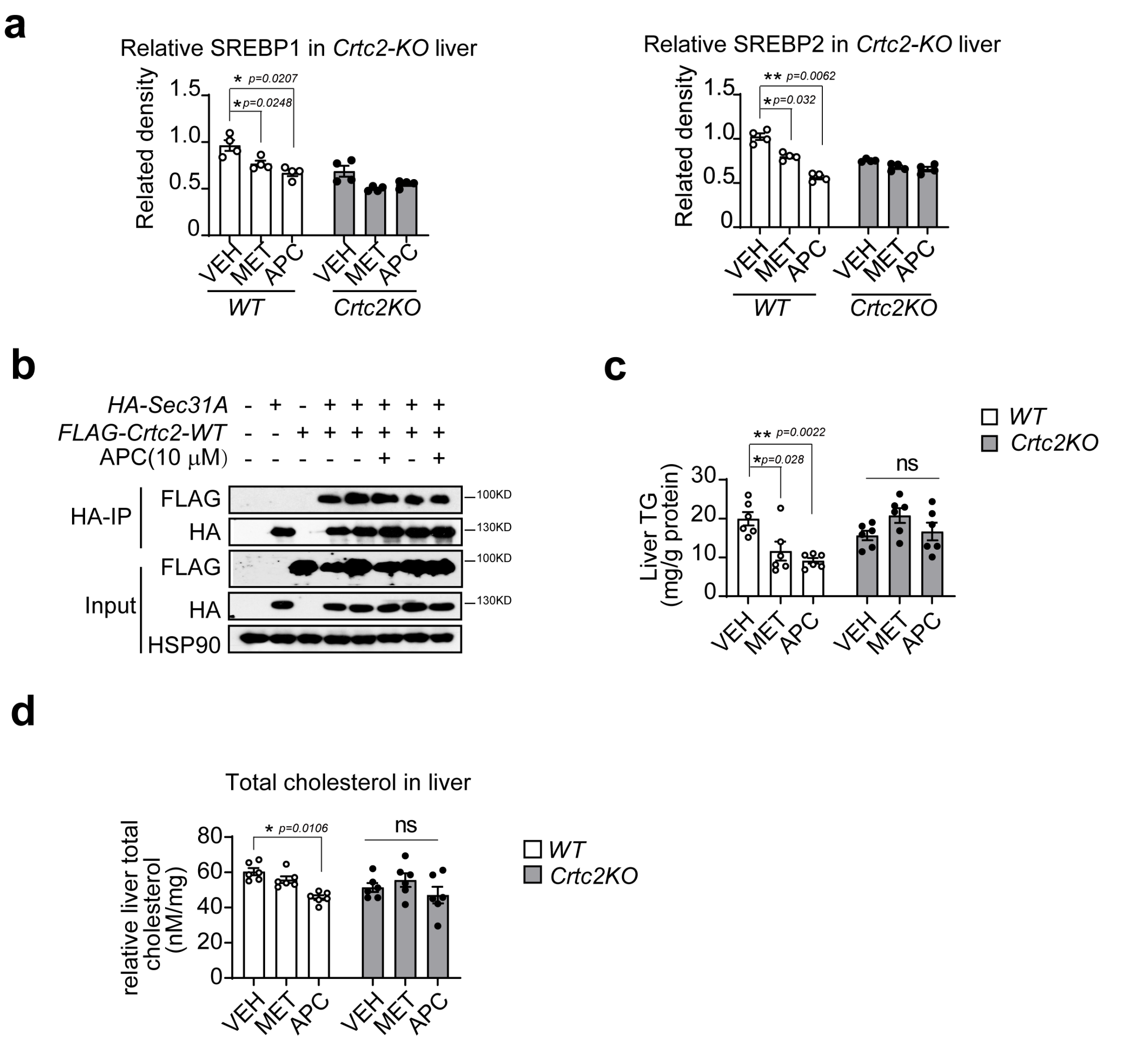

Supplementary Figure 5. APC decreases SREBP1 and 2 protein levels but does not impair interaction between CRTC2 and SEC31A

a) The relative SREBP1 (left) and SREBP2 (right) protein levels in the liver of *Crtc2*KO mice was induced by a high fat diet for 8 weeks. *Crtc2*KO mice and wild type littermates were administrated VEH (vehicle control), MET (metformin, 200 mg/kg) or APC (20 mg/kg) for 3 weeks, and fasted 8 h before anesthesia. Data are represented as mean ± SEM (n=4). *, $p < 0.05$; **, $p < 0.01$; p values were determined by two-way ANOVA followed Bonferroni's multiple comparisons test.

b) Immunoprecipitation of SEC31A from HEK293 cell lysates with overexpressed *HA-Sec13A* or *FLAG-Crtc2-WT*. APC (10 μM) was added to the IP mixture overnight. One of two independent experiments is shown here.

c) Triglyceride (TG) concentration and （**d**） total cholesterol levels in the liver of *Crtc2*KO mice administered MET and APC as in **(a)**. Data are represented as mean ± SEM (n=6 per group). *, $p < 0.05$; **, $p < 0.01$; p values were determined by two-way ANOVA followed Bonferroni's multiple comparisons test. Source data for this figure are provided as a Source data file.

Supplementary Fig. 6

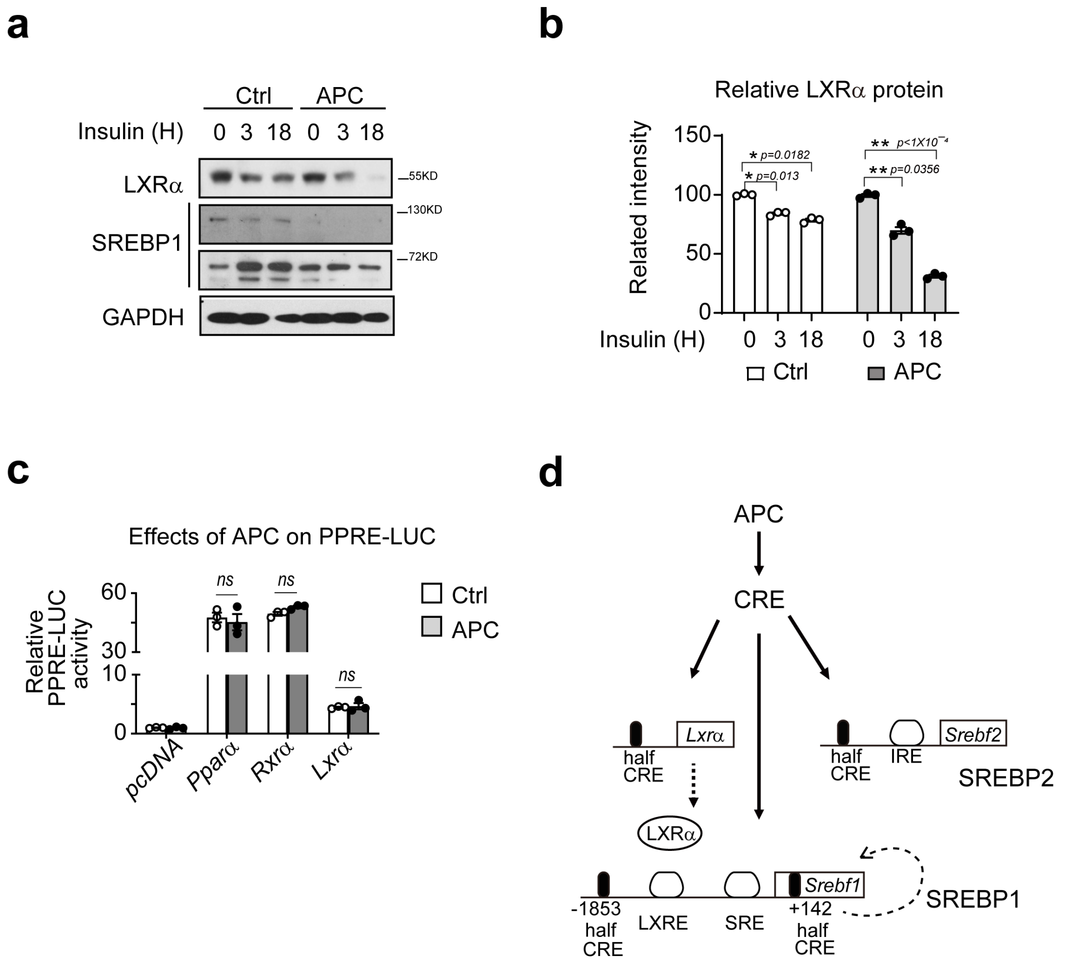

Supplementary Figure 6. APC inhibited transcription induced by half-CRE in the promoters of LXRα and SREBP2.

a) Immunoblot of LXRα protein levels in primary hepatocytes stimulated with insulin (100 nM) for indicated times (0–18 h). One presentative result from three experiments is shown here. Relative LXRα protein level normalized to GAPDH shown as a bar graph **(b).** Data are represented as mean ± SEM (n=3 per treatment). *, $p < 0.05$; **, $p < 0.01$; p values were determined by two-way ANOVA repeated measures followed Bonferroni's multiple comparisons test.

c) Luciferase reporter activity of *PPRE-LUC* (PPAR response element driven luciferase reporter) in HEK293T cell transfected with expression plasmids for *Pparα*, *Rxrα* or *Lxrα* with 8-h incubation of APC (10 μM). One of three independent experiments presented here. Data are represented as mean ± SEM. (n=3 per treatment). *, $p < 0.05$; **, $p < 0.01$; p values were determined by two-way ANOVA followed Bonferroni's multiple comparisons test.

d) Schematic of APC inhibiting SREBP1 expression via the CRE-LXRα-LXRE axis, and reducing SREBP2 expression by blocking half-CRE directly. Source data for this figure are provided as a Source data file.

Supplementary Fig. 7

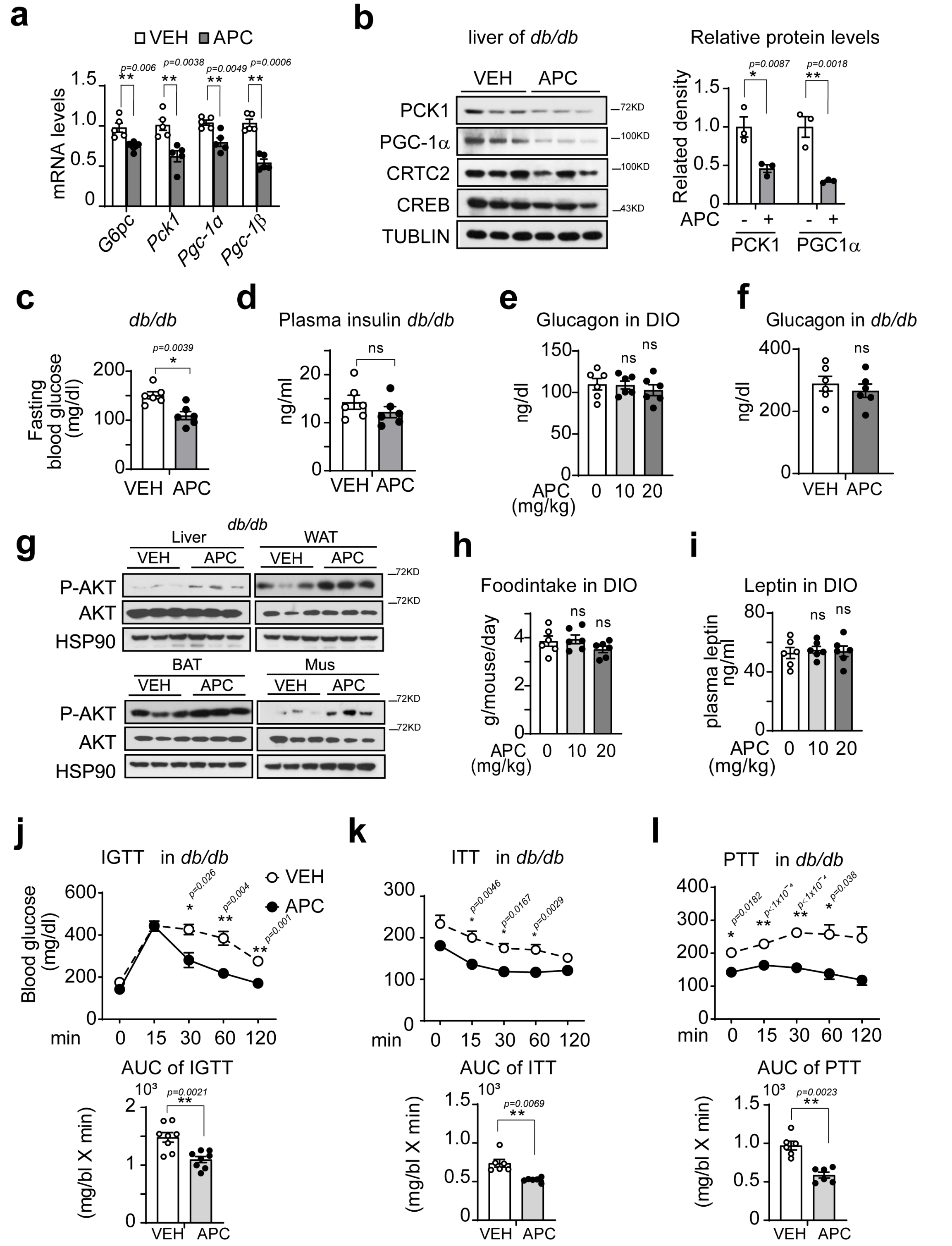

Supplementary Figure 7. APC increases insulin sensitivity in *db/db* mice.

db/db mice were continuously *i.p.* injected synthesized APC (20 mg/kg) or control vehicle (VEH) one time daily for 5 weeks (n = 5–7).

a) Quantitative PCR analysis of *G6pc, Pck1, Pgc-1α* and *Pgc-1β* gene expression in the liver of treated *db/db* mice. Data are represented as mean ± SEM (n=5 per group). *, $p < 0.05$; **, $p < 0.01$; p values were determined by unpaired two-tailed multiple t test with two-stage linear step-up procedure, each gene was analyzed individually, without assuming a consistent SD.

b) Immunoblotting of endogenous protein levels of PCK1, PGC-1α, CREB and CRTC2 in the livers of 16-h-fasted *db/db* mice *i.p.* administered APC (20 mg/kg) or vehicle (VEH) one time daily for 5 weeks. One presentative result from two experiments is shown here (left), and relative PCK1 and PGC-1α normalized to TUBULIN levels is presented as a bar graph (right). Data are represented as mean ± SEM (n=3 per treatment). *, $p < 0.05$; **, $p < 0.01$; p values were determined by two-way ANOVA followed Bonferroni's multiple comparisons test.

c) Fasting blood glucose levels and (***d***) plasma insulin of treated *db/db* mice (**d**) (n=6 per group). Data are represented as mean ± SEM. ns, $p > 0.05$; *, $p < 0.05$; **, $p < 0.01$; p values were determined by unpaired two-tailed t test with Welch's correction.

e) Plasma glucagon of DIO mice as in Fig. **7(d)** and **(f)** glucagon level in treated *db/db* mice. Data are represented as mean ± SEM (n=6 per group). *, $p < 0.05$; **, $p < 0.01$; p values were determined by one-way ANOVA followed Dunnett's test in (**e**), or by unpaired two-tailed t test with Welch's correction in (**f**).

g) Immunoblotting for phosphorylated AKT and total AKT in tissues from the liver, white adipose (WAT), brown fat (BAT) and muscle (Mus) of treated *db/db* mice. One presentative result from two experiments is shown here (n=3).

h) Daily food intake and plasma leptin level **(i)** of DIO mice stimulated with high fat diet for 13 weeks and 2 weeks of APC injection (n=6 per group). One of two independent experiments is shown here. Data are represented as mean ± SEM. *, $p < 0.05$; **, $p < 0.01$; p values were determined by one-way ANOVA followed Dunnett's test.

j) Injected glucose tolerance test (IGTT), (**k**) insulin tolerance test (ITT) and (**l**) pyruvate tolerance test (PTT) of treated *db/db* mice. The results of area under curve (AUC) are shown at the bottom of each test curve. Data are represented as mean ± SEM (n=6-8 per group). *, $p < 0.05$; **, $p < 0.01$; p values of curves were determined by two-way ANOVA followed Bonferroni's multiple comparisons test, and p values of AUC were determined by unpaired two-tailed t test with Welch's correction. Source data for this figure are provided as a Source data file.

Supplementary Fig.8

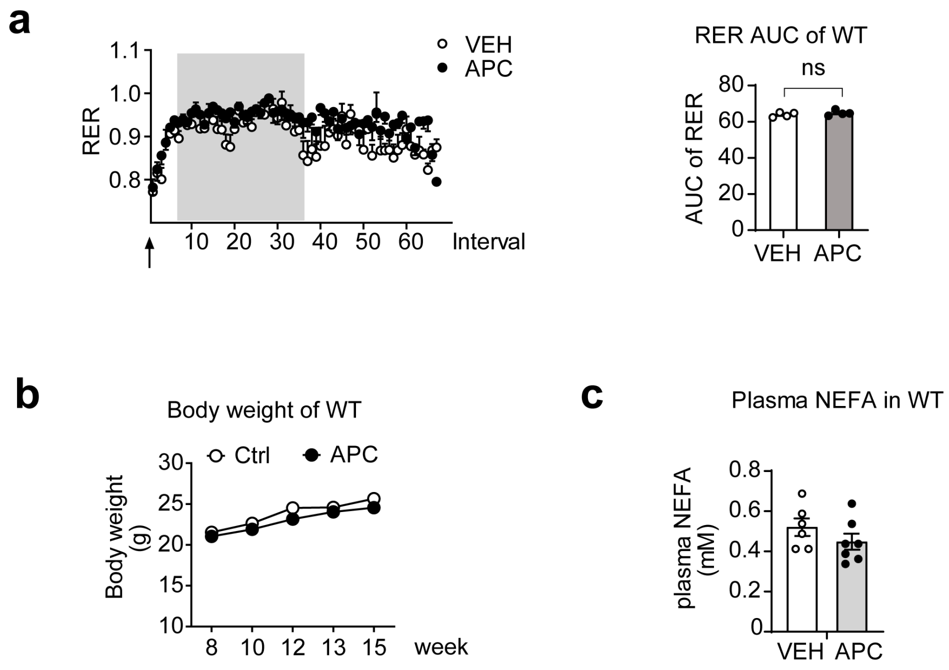

Supplementary Figure 8. APC had no impact on lean wild type mice.

Lean mice (wild C57) were *i.p.* injected with APC (20 mg/kg) or vehicle (VEH) one time daily for 3 weeks (n=7).

a) Twenty-four hours respiratory exchange ratio (RER), and a time course of RER curves (left) was analyzed by AUC analysis (right) for these lean mice. Data are represented as mean ± SEM. (n=4 per group). ns, $p > 0.05$; *, $p < 0.05$; **, $p < 0.01$; p values were determined by two-way ANOVA followed Bonferroni's multiple comparisons test, or by unpaired two tailed t-test with Welch's correction in AUC analysis of RER.

b) Body weight curves and (**c**) Plasma NEFA in lean mice. One of two independent experiments is shown here. Data are represented as mean ± SEM. (n=7 per group). ns, $p > 0.05$; *, $p < 0.05$; **, $p < 0.01$; p determined by two-way ANOVA followed Bonferroni's multiple comparisons test for (**b**),

or by unpaired two tailed *t*-test with Welch's test for (**c**). Source data for this figure are provided as a Source data file.

Supplementary Figure 9. Synthesis and identification of A57

a) Synthetic route to compound **A57**. (a) malonic acid, piperidine, pyridine, reflux, 92%; (b) methanol, conc. H_2SO_4, argon, reflux, 98%; (c) $PhB(OH)_2$, $Pd(PPh_3)_4$, Na_2CO_3, toluene-methanol-water, 80°C, 89%; (d) LiOH, rt, 97%; (e) (*S*)-4-phenyloxazolidin-2-one, pivaloyl chloride, Et_3N, LiCl, rt, 90%. (f) (*R*)-4-phenyloxazolidin-2-one, pivaloyl chloride, Et_3N, LiCl, rt, 89%. (g) (*rac*)-4-phenyloxazolidin-2-one, pivaloyl chloride, Et_3N, LiCl, rt, 80%.

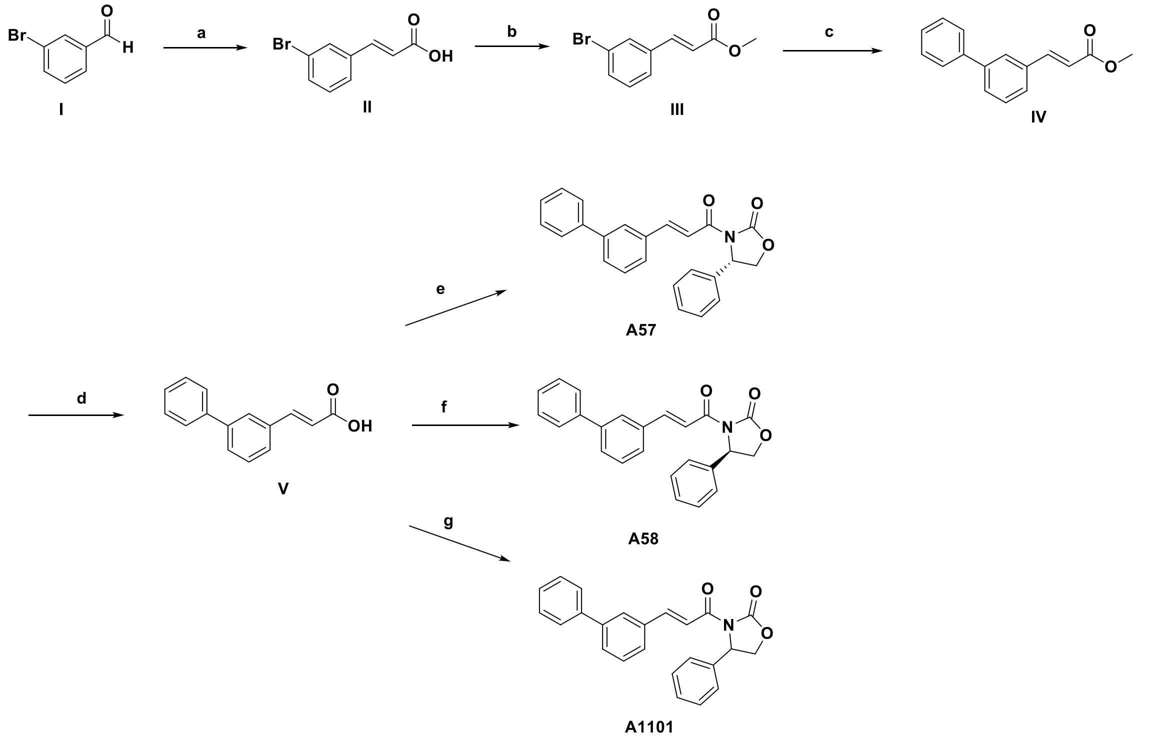

Synthesis compound A57

Synthesis of (*E*)-3-(3-bromophenyl) acrylic acid **II**

A suspension of 3-bromobenzaldehyde **I** (1.00 g, 5.40 mmol), malonic acid (2.53 g, 24.32 mmol), and pyridine-piperidine (70:1, v/v, 70 mL) was heated with reflux for 24 h. After cooling, the mixture was adjusted to pH 1 by the addition of 1 *N* HCl. The resulting precipitate was collected by filtration, washed with water, and then dried *in vacuo* to give (*E*)-3-(3-bromophenyl) acrylic acid **II** as colorless crystals

(1.13 g, 92%). ^{1}H NMR (400 MHz, DMSO-d_6) δ 12.53 (s, 1H), 7.95 (t, J = 1.9 Hz, 1H), 7.72 (d, J = 7.8 Hz, 1H), 7.61 (dd, J = 7.8, 1.8 Hz, 1H), 7.57 (d, J = 16.1 Hz, 1H), 7.38 (t, J = 7.9 Hz, 1H), 6.62 (d, J = 16.0 Hz, 1H). ^{13}C NMR (125 MHz, DMSO-d_6) δ 167.8, 142.7, 137.3, 133.2, 131.4, 131.2, 127.6, 122.7, 121.4. ESI/LRMS: (m/z) 224.9, 226.9 $[M-H]^-$. ESI/HRMS: (m/z) calcd for $C_9H_6BrO_2$ $[M-H]^-$ 224.9557, yielded 224.9555.

Synthesis of methyl (*E*)-3-(3-bromophenyl) acrylate **III**

Acrylic acid **II** (1.00 g, 4.40 mmol) and anhydrous methanol (25 mL) were added to a dried round flask, followed by the dropwise addition of concentrated sulfuric acid (0.26 mL, 4.84 mmol). The resulting mixture was charged and protected with argon and refluxed overnight. The reaction mixture was allowed to cool to room temperature. After the solvent was removed *in vacuo*, the residue was added to EtOAc and H_2O, and the phases were separated. The aqueous layer was extracted again with EtOAc, then the combined organic layers were washed with H_2O, Na_2CO_3 (aq.), and brine, dried over Na_2SO_4, filtered and then the solvent was removed *in vacuo* to afford methyl (*E*)-3-(3-bromophenyl) acrylate **III** as a colorless solid (1.04 g, 98%). ^{1}H NMR (400 MHz, DMSO-d_6) δ 7.98 (ddd, J = 9.1, 3.7, 1.9 Hz, 1H), 7.78 – 7.70 (m, 1H), 7.68 – 7.57 (m, 2H), 7.37 (tdd, J = 7.5, 5.0, 2.3 Hz, 1H), 6.79 – 6.68 (m, 1H), 3.73 (d, J = 1.6 Hz, 3H).

Synthesis of methyl (*E*)-3-([1,1'-biphenyl]-3-yl) acrylate **IV**

A mixture of phenylboronic acid (424.8 mg, 3.48 mmol), sodium carbonate (527.6 mg, 4.98 mmol), and methyl (*E*)-3-(3-bromophenyl) acrylate **III** (600.0 mg, 2.49 mmol) in toluene (6 mL), MeOH (3

mL) and water (6 mL) was added to tetrakis-(triphenylphosphine) palladium (86.3 mg, 0.75 mmol) at room temperature. The resulting mixture was heated to 80°C with stirring for 10 h under an argon atmosphere. Then the reaction mixture was cooled to room temperature and extracted with EtOAc. The extract was dried over Na_2SO_4, and concentrated. The residue was purified by silica gel column chromatography (hexane: EtOAc = 30: 1) to give methyl (*E*)-3-([1,1'-biphenyl]-3-yl) acrylate **IV** as a white powder (528.5 mg, 89%). ^{1}H NMR (400 MHz, DMSO-d_6) δ 8.04 (d, J = 2.0 Hz, 1H), 7.79 – 7.72 (m, 5H), 7.56 – 7.46 (m, 3H), 7.43 – 7.37 (m, 1H), 6.80 (dd, J = 16.1, 0.9 Hz, 1H), 3.75 (s, 3H). ^{13}C NMR (125 MHz, $CDCl_3$) δ 166.9, 143.1, 136.5, 133.1, 130.7, 130.4, 126.7, 123.0, 119.3, 51.8. EI/LRMS: (m/z) 240. EI/HRMS: (m/z) calcd for $C_{10}H_9O_2Br$ 239.9780, found 239.9784.

Synthesis of (*E*)-3-([1,1'-biphenyl]-3-yl) acrylic acid **V**

Acrylate **IV** (500.0 mg, 2.10 mmol) was dissolved in MeOH-H_2O (1:1, v/v), then LiOH (100.5 mg, 4.20 mmol) was added, and the resulting mixture was stirred at room temperature overnight. After completion, 15 mL of heptane was added to the reaction mixture with 15 min stirring. Then the phases were separated, and the aqueous layer was adjusted to pH 4 by the addition of a 10% citric acid aqueous solution. The resulting precipitate was collected by filtration, washed with water, and then dried *in vacuo* to give (*E*)-3-([1,1'-biphenyl]-3-yl) acrylic acid **V** as a white powder (456.4 mg, 97%).^{1}H NMR (400 MHz, DMSO-d_6) δ 12.42 (s, 1H), 7.99 (d, J = 2.2 Hz, 1H), 7.78 – 7.66 (m, 5H), 7.55 – 7.46 (m, 3H), 7.43 – 7.37 (m, 1H), 6.68 (d, J = 16.1 Hz, 1H). ^{13}C NMR (125 MHz, DMSO-d_6) δ 167.6, 143.8, 140.8, 139.4, 134.9, 129.5, 128.9 (2CH), 128.4, 127.7, 127.0, 126.8 (2CH), 126.6,

119.7. ESI/LRMS: (m/z) 223.1 [M-H]$^-$. ESI/HRMS: (m/z) calcd for $C_{15}H_{11}O_2$ [M-H]$^-$ 223.0765, found 223.0763.

Synthesis of (*S,E*)-3-(3-([1,1'-biphenyl]-3-yl)acryloyl)-4-phenyloxazolidin-2-one **A57**

The acrylic acid **V** (400.0 mg, 1.78 mmol), Et_3N (0.62 mL, 4.46 mmol) and anhydrous dichloromethane were added to a dried flask, and the solution was stirred under an argon atmosphere at -78°C, and pivaloyl chloride (0.33 mL, 2.68 mmol) was then added. The resulting mixture was allowed to warm to room temperature and stirred for 1 h. Then, the reaction mixture was cooled to -5°C, and (*S*)-4-phenyloxazolidin-2-one (291.1 mg, 1.78 mmol) and LiCl (75.6 mg, 1.78 mmol) were added. The resulting mixture was warmed to room temperature and stirred overnight. After completion, the reaction mixture was quenched by water and extracted with dichloromethane, the organic layer was washed with brine, dried over Na_2SO_4, and concentrated *in vacuo*. The residue was purified by silica gel column chromatography (DCM: MeOH = 200: 1) to give (*S,E*)-3-(3-([1,1'-biphenyl]-3-yl)acryloyl)-4-phenyloxazolidin-2-one **A57** as a white powder (595.7 mg, yield 90%, chemical purity 99.7%, *ee*> 99.9%). m.p. 138.6-140.2 °C, $[\alpha]^{20}_D$ = -6 (c 0.100 g/100mL, $CHCl_3$), ^{1}H NMR (400 MHz, DMSO-d_6) δ 8.00 – 7.88 (m, 2H), 7.81 – 7.66 (m, 5H), 7.56 (t, *J* = 7.7 Hz, 1H), 7.53 – 7.47 (m, 2H), 7.45 – 7.33 (m, 6H), 5.61 (dd, *J* = 8.6, 3.9 Hz, 1H), 4.87 – 4.78 (m, 1H), 4.27 – 4.20 (m, 1H). ^{13}C NMR (125 MHz, DMSO-d_6) δ 164.0 (C=O), 153.9 (O-C=O), 144.5 (HC=), 141.0 (C-1'), 139.7 (C-1), 139.4 (C-1 of (*S*)-Phenyl), 134.9 (C-3), 129.7 (C-2), 129.0 (C-5, C-3', C-5'), 128.8 (C-3 and C-5 of (*S*)-Phenyl), 128.0 (C-6), 127.8 (C-4'), 127.0 (C-4 of (*S*)-Phenyl), 126.8 (C-2', C-6'), 126.7 (C-4), 125.85 (C-2 and C-6 of

(*S*)-Phenyl), 118.2 (=CH), 70.2 (CH_2-O), 57.2 (CH-N). ESI/LRMS: (*m/z*) 370.1 $[M+H]^+$, 391.9 $[M+Na]^+$. ESI/HRMS: (*m/z*) calcd for $C_{24}H_{20}NO_3^+$ $[M+H]^+$ 370.1438, found 370.1448. The chemical purity was determined by HPLC with an Agilent Extend-C18 column (5 μm, 4.6×150 mm) (MeOH/H_2O = 70/30, λ = 254 nm, 1.0 mL/min), purity 99.7%, t = 26.339 min. The *ee* value was determined by HPLC with a Chiralpak IA column (*n*-hexane/*i*-PrOH = 70/30, λ = 254 nm, 1.0 mL/min), t_{major} = 15.400 min, *ee* > 99.9%.

Synthesis of the compound A58

Synthesis of (*R,E*)-3-(3-([1,1'-biphenyl]-3-yl)acryloyl)-4-phenyloxazolidin-2-one **A58**

The compound **A58**, (*R,E*)-3-(3-([1,1'-biphenyl]-3-yl)acryloyl)-4-phenyloxazolidin-2-one, was prepared from **V** and (*R*)-4-phenyloxazolidin-2-one according to the procedure described for compound **A57** in 89% yield as a white powder, chemical purity 99.5%, *ee* > 99.9%. $[\alpha]^{20}_D$ = +6 (c 0.100 g/100mL, $CHCl_3$), ^1H NMR (400 MHz, DMSO-d_6) δ 7.96 – 7.89 (m, 2H), 7.81 – 7.67 (m, 5H), 7.56 (t, *J* = 7.7 Hz, 1H), 7.50 (dd, *J* = 8.2, 6.9 Hz, 2H), 7.44 – 7.32 (m, 6H), 5.61 (dd, *J* = 8.6, 3.9 Hz, 1H), 4.82 (t, *J* = 8.7 Hz, 1H), 4.23 (dd, *J* = 8.6, 3.9 Hz, 1H). ^{13}C NMR (125 MHz, DMSO-d_6) δ 164.0 (C=O), 153.9 (O-C=O), 144.5 (HC=), 141.0 (C-1’), 139.7 (C-1), 139.4 (C-1 of (*R*)-Phenyl), 134.9 (C-3), 129.7 (C-2), 129.0 (C-5, C-3’, C-5’), 128.8 (C-3 and C-5 of (*R*)-Phenyl), 128.0 (C-6), 127.8 (C-4’), 127.0 (C-4 of (*R*)-Phenyl), 126.8 (C-2’, C-6’), 126.7 (C-4), 125.85 (C-2 and C-6 of (*R*)-Phenyl), 118.2 (=CH), 70.2 (CH_2-O), 57.2 (CH-N). LRMS (ESI, *m/z*): 370.1 $[M+H]^+$, 391.9 $[M+Na]^+$; HRMS (ESI) cacld for $C_{24}H_{19}NNaO_3$ $[M+Na]^+$: 392.1257, found: 392.1264. The chemical purity was determined by HPLC with an Agilent Extend-C18 column (5 μm, 4.6×150 mm) (MeOH/H_2O = 80/20, λ = 254 nm, 1.0

mL/min), purity 99.0%, t = 6.543 min. The *ee* value was determined by HPLC with a Chiralpak IA column (*n*-hexane/*i*-PrOH = 70/30, λ = 254 nm, 1.0 mL/min), t_{major} = 51.571 min, *ee* > 99.9%.

Synthesis of the compound A1101

Synthesis of (*rac,E*)-3-(3-([1,1'-biphenyl]-3-yl)acryloyl)-4-phenyloxazolidin-2-one **A1101**

The compound **A1101**, (*rac,E*)-3-(3-([1,1'-biphenyl]-3-yl)acryloyl)-4-phenyloxazolidin-2-one, was prepared from **V** and (*rac*)-4-phenyloxazolidin-2-one according to the procedure described for compound **A57** in 80% yield as a white powder, chemical purity 99.0%, *ee* = 0.1%. ^{1}H NMR (500 MHz, DMSO-d_6) δ 7.95 – 7.86 (m, 2H), 7.81 – 7.65 (m, 5H), 7.57 (t, *J* = 7.7 Hz, 1H), 7.50 (dd, *J* = 8.3, 7.1 Hz, 2H), 7.45 – 7.30 (m, 6H), 5.61 (dd, *J* = 8.7, 3.9 Hz, 1H), 4.82 (t, *J* = 8.7 Hz, 1H), 4.23 (dd, *J* = 8.7, 3.9 Hz, 1H). ^{13}C NMR (125 MHz, DMSO-d_6) δ 164.0 (C=O), 153.9 (O-C=O), 144.5 (HC=), 141.0 (C-1’), 139.7 (C-1), 139.4 (C-1 of (*rac*)-Phenyl), 134.9 (C-3), 129.7 (C-2), 129.0 (C-5, C-3’, C-5’), 128.8 (C-3 and C-5 of (*rac*)-Phenyl), 128.0 (C-6), 127.8 (C-4’), 127.0 (C-4 of (*rac*)-Phenyl), 126.8 (C-2’, C-6’), 126.7 (C-4), 125.85 (C-2 and C-6 of (*rac*)-Phenyl), 118.2 (=CH), 70.2 (CH_2-O), 57.2 (CH-N). LRMS (ESI, *m/z*): 370.1 $[M+H]^+$, 391.9 $[M+Na]^+$; HRMS (ESI) cacld for $C_{24}H_{20}NO_3$ $[M+H]^+$: 370.1438, found: 370.1445. The chemical purity was determined by HPLC with an Agilent Extend-C18 column (5 μm, 4.6×150 mm) (MeOH/H_2O = 80/20, λ = 254 nm, 1.0 mL/min), purity 100.0%, t = 6.274 min. The *ee* value was determined by HPLC with a Chiralpak IA column (*n*-hexane/*i*-PrOH = 70/30, λ = 254 nm, 1.0 mL/min), t_1 = 15.481 min, t_2 = 50.245 min, *ee* = 0.1%.

b) ¹H NMR spectrum of compound **A57**, **A58** and **A1101**

¹H NMR spectrum of compound **A57** (400 MHz, DMSO-d_6)

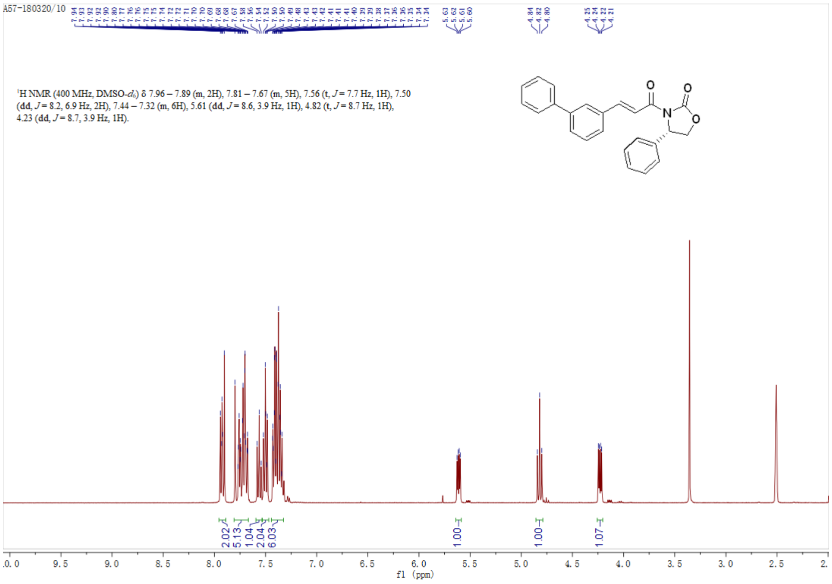

¹H NMR spectrum of the compound **A58** (400 MHz, DMSO-d_6)

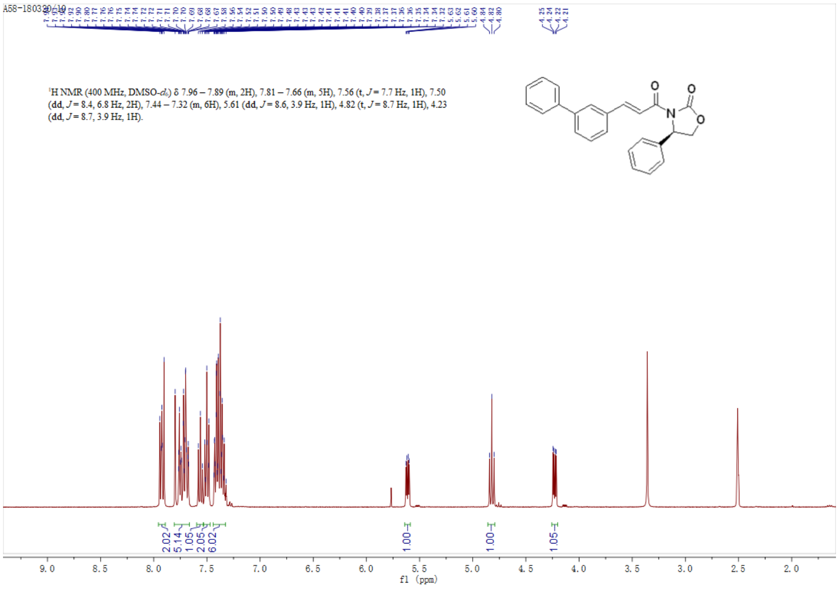

^{1}H NMR spectrum of the compound **A1101** (500 MHz, DMSO-d_6)

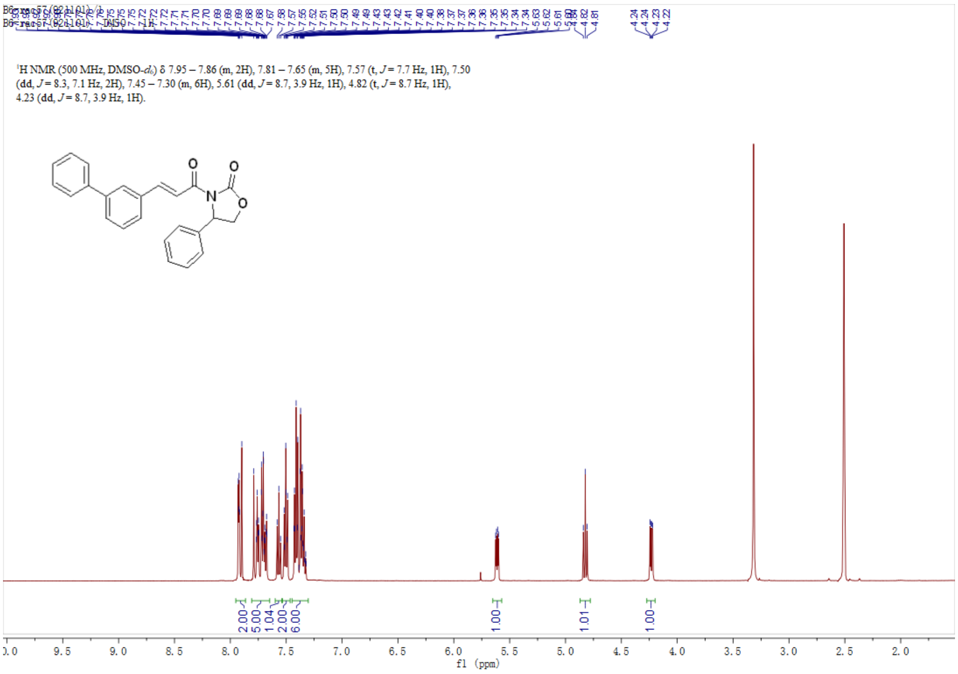

c) ^{13}C NMR spectrum of compound **A57**, **A58** and **A1101**

^{13}C NMR spectrum of compound **A57** (125 MHz, DMSO-d_6)

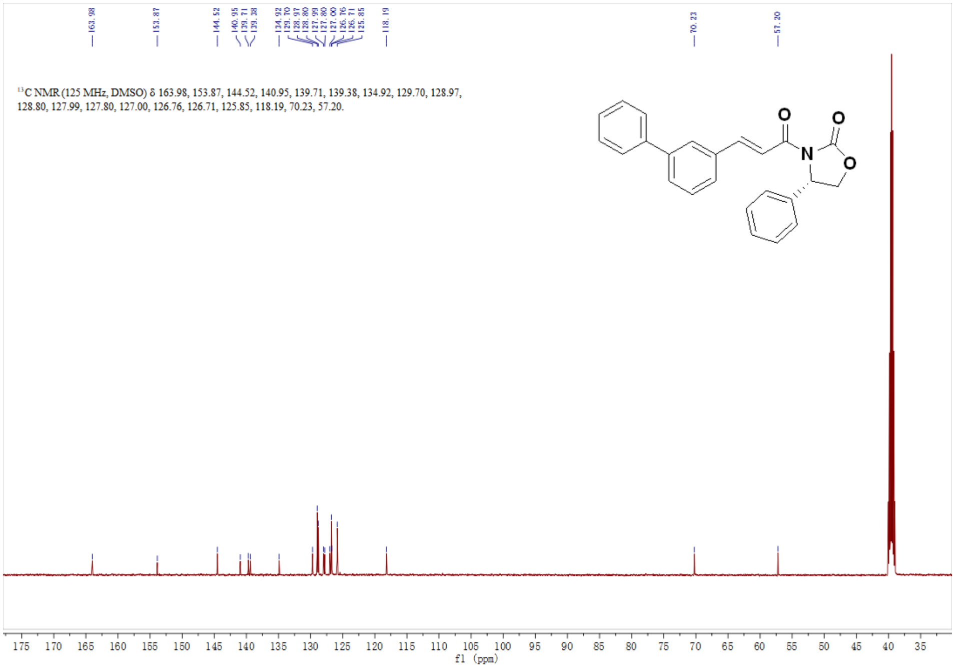

^{13}C NMR spectrum of the compound **A58** (125 MHz, DMSO-d_6)

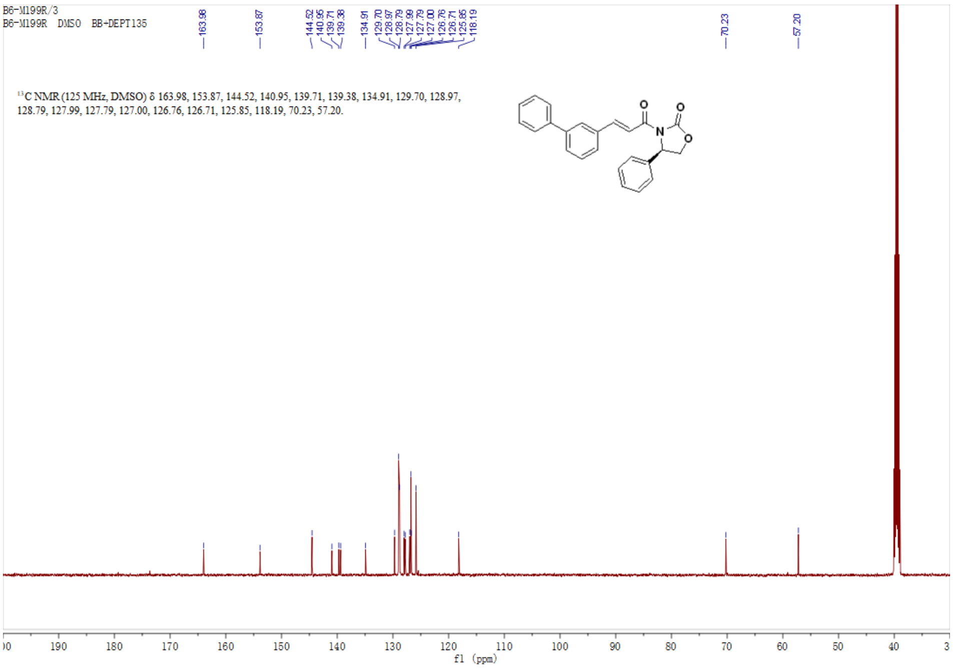

^{13}C NMR spectrum of the compound **A1101** (125 MHz, DMSO-d_6)

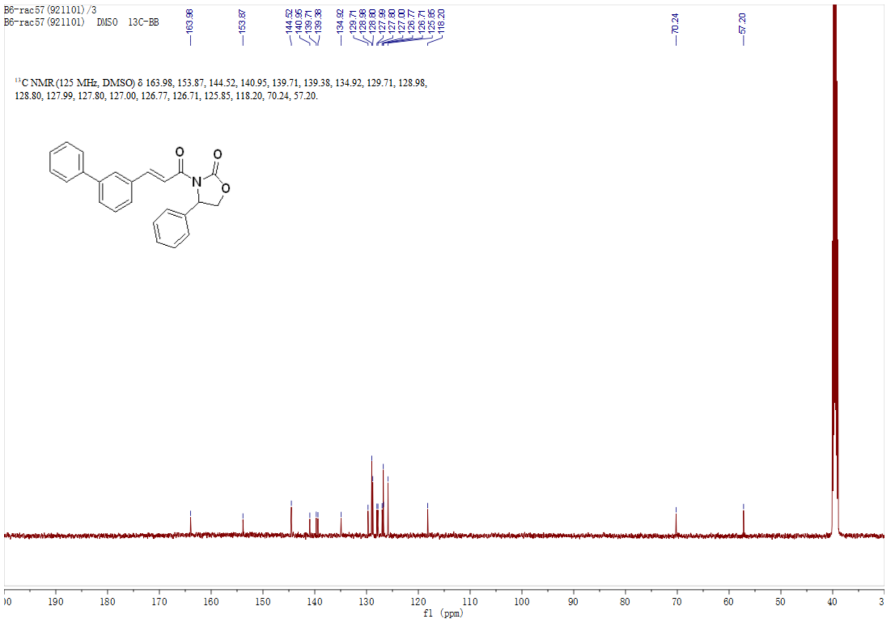

d) HPLC spectrum of compound **A57**, **A58** and **A1101** (chemical purity determination)

Method A: Agilent Extend-C18 column (5 µm, 4.6×150 mm) (MeOH/H_2O = 70/30, λ = 254 nm, 1.0 mL/min), **A57** purity 99.7%.

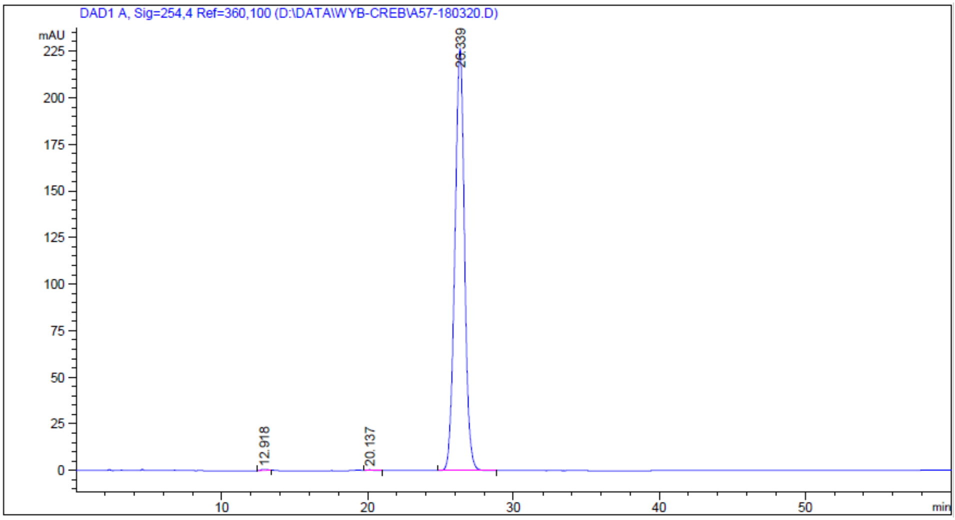

Peak #	RetTime [min]	Type	Width [min]	Area [mAU*s]	Height [mAU]	Area %
1	12.918	BB	0.4412	15.57250	5.14396e-1	0.1493
2	20.137	VB	0.4645	14.09288	4.29803e-1	0.1351
3	26.339	BB	0.7044	1.04006e4	226.20932	99.7156

Totals : 1.04303e4 227.15352

HPLC spectrum of the compound **A58** (chemical purity determination)

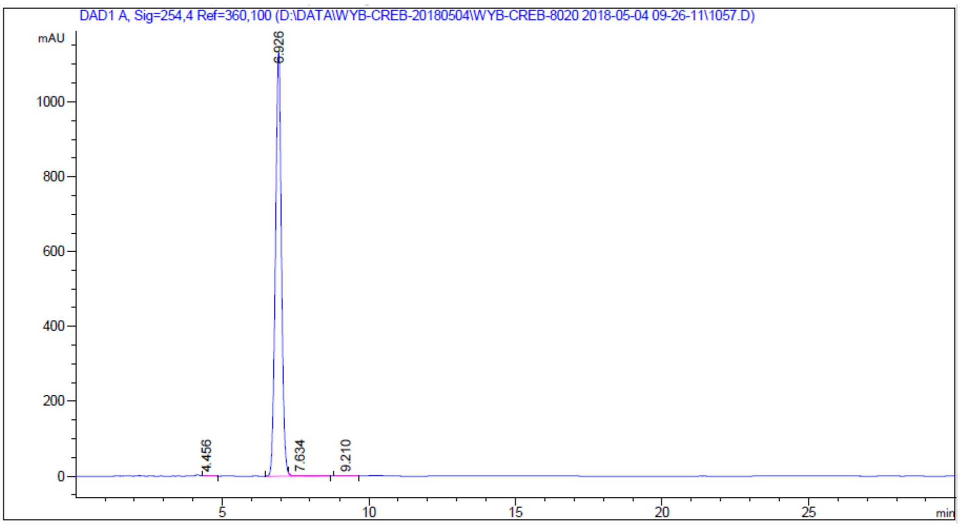

Signal 1: DAD1 A, Sig=254,4 Ref=360,100

Peak #	RetTime [min]	Type	Width [min]	Area [mAU*s]	Height [mAU]	Area %
1	4.456	VB	0.1615	10.42765	9.84879e-1	0.0642
2	6.926	BV R	0.2204	1.61569e4	1133.11365	99.5074
3	7.634	VB E	0.3716	51.74415	1.96258	0.3187
4	9.210	BB	0.3027	17.80532	8.85208e-1	0.1097
Totals :				1.62369e4	1136.94632	

HPLC spectrum of the compound **A58** Method B: Agilent Extend-C18 column (5 μm, 4.6×150 mm) (MeOH/H_2O = 80/20, λ = 254 nm, 1.0 mL/min), **A58** purity 99.5%.

HPLC spectrum of the compound **A1101** (chemical purity determination)

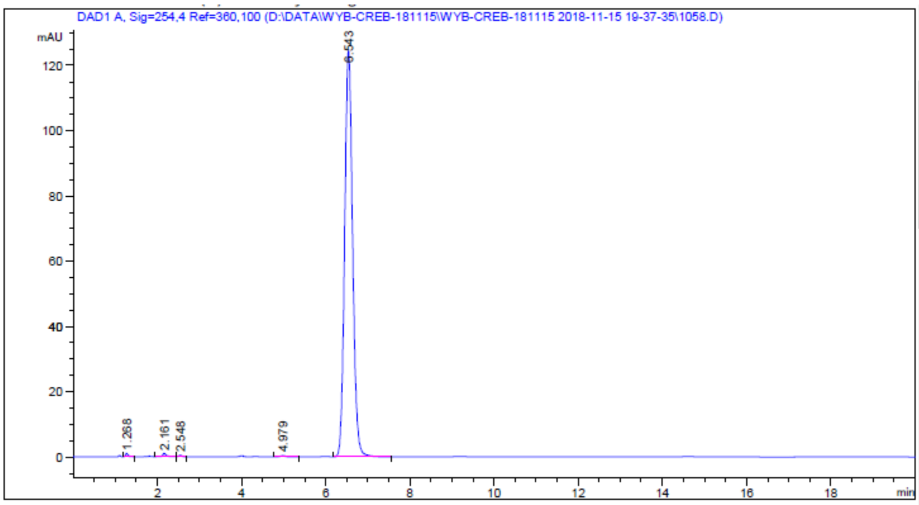

Signal 1: DAD1 A, Sig=254,4 Ref=360,100

Peak #	RetTime [min]	Type	Width [min]	Area [mAU*s]	Height [mAU]	Area %
1	1.268	VV R	0.0674	4.69879	1.01277	0.2845
2	2.161	VV R	0.0872	6.37240	1.01949	0.3858
3	2.548	VB	0.0846	2.37255	4.15400e-1	0.1436
4	4.979	BB	0.1381	2.68904	3.01236e-1	0.1628
5	6.543	BB	0.2035	1635.71960	124.32829	99.0234
Totals :				1651.85237	127.07719	

HPLC spectrum of the compound **A1101** Method B: Agilent Extend-C18 column (5 μm, 4.6×150 mm) ($MeOH/H_2O$ = 80/20, λ = 254 nm, 1.0 mL/min), **A1101** purity 100.0%

(E) The configuration of compounds **A57 (A)** and **A58 (B).**

The *S*-configured enantiomer **A57** (IC_{50} = 0.74 μM) was more effective than the *R*-configured compound **A58** (IC_{50} =73.5 μM) and the racemate **A1101** (IC_{50} =3.56 μM). These results suggest that the configuration of the phenyl group is related to the inhibitory activity. The configuration of compounds **A57** and **A58** was shown in suppl. Fig. 9e. The different configuration of the phenyl group in these two compounds explains why compound **A57** has better inhibitory activity than **A58**. For the *S*-configured compound **A57**, phenyl group in compound **A57** may occupy the active pocket of protein CREB, which increase the inhibitory activity. Consistent with the poorer inhibitory activity to CREB/CRTC2

interaction, compound **A58** exhibits a different configuration. It is possible that the steric bulk of the phenyl group in compound **A58** hinder interaction between the compound **A58** and the protein CREB, which reduced the inhibitory activity.

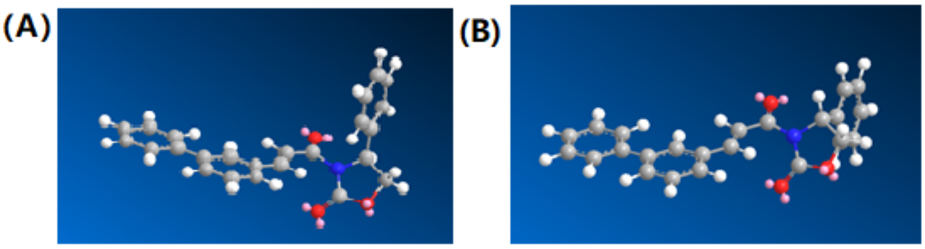

Sup. Figure 9e. The configuration of compounds **A57 (A)** and **A58 (B).**

Supplementary Fig.10

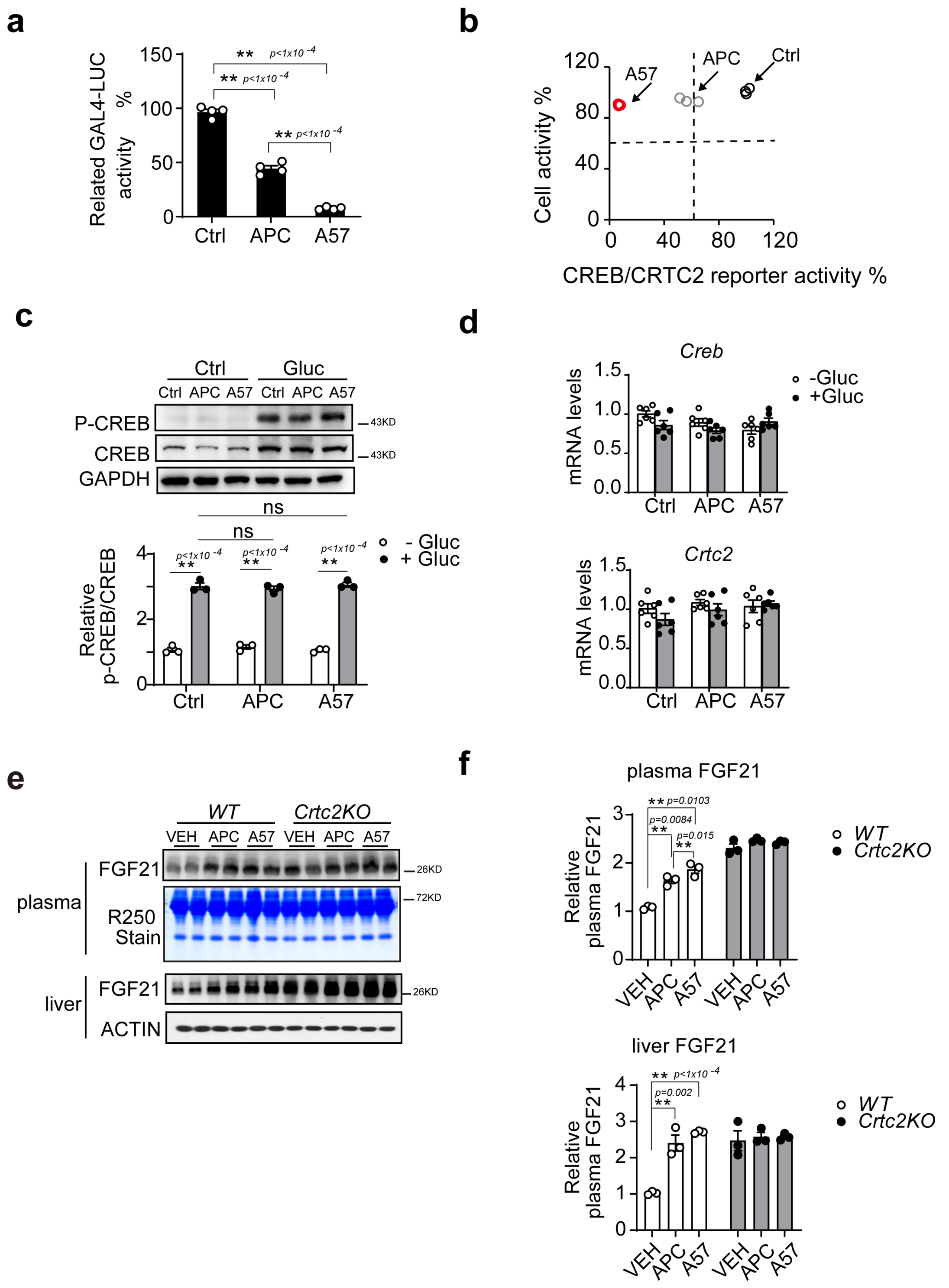

Supplementary Figure 10. A57 is novel inhibitor of the CREB/CRTC2 protein complex.

a) The inhibitory-activity of **A57** as determined by two-hybrid reporter assay. HEK293T cells were co-transfected with CREB/CRTC2 two-hybrid reporter system plasmids, following incubation with indicated small molecules (10 μM) overnight before luciferase reporter assays (*n*=4 per treatment). One of three independent experiments is shown here. Data are represented as mean ± SEM. *, $p < 0.05$; **, $p < 0.01$; *p* values were determined by one-way ANOVA followed Dunnett's multiple comparisons test.

b) Combinatorial analysis of the cell toxicity and inhibitory activity of **A57**. The CREB/CRTC2-two hybrid reporter activity (as a normalized percentage) is the X-axis, and the cell activity tested by MTT assay (as a normalized percentage) is the Y-axis. n=3.

c) Immunoblot of P-CREB and CRTC2 de-phosphorylation in primary hepatocytes incubated with **A57** and APC (10 μM) 1-h prior to glucagon (100 nM) stimulation for 30 min. One result from three experiments is shown here (top), and relative P-CREB normalized by CREB is presented as a bar graph (bottom, *n*=3). Data are represented as mean ± SEM. ns, $p > 0.05$; *, $p < 0.05$; **, $p < 0.01$; *p* values were determined by two-way ANOVA followed Bonferroni's multiple comparisons test.

d) mRNA levels of *Creb* (top) and *Crtc2* (bottom) in primary hepatocytes incubated with **A57** (10 μM) 1-h prior to glucagon (100 nM) stimulation for 4-h (*n*=6 per treatment). Data are represented as mean ± SEM. ns, $p > 0.05$; *, $p < 0.05$; **, $p < 0.01$; *p* values were determined by two-way ANOVA followed Bonferroni's multiple comparisons test.

e) Immunoblotting of plasma and liver FGF21 protein in *Crtc2*KO mice, which induced by a high fat diet for 13 weeks then orally administered APC (20 mg/kg), **A57** (20 mg/kg) or vehicle (VEH) for

3 weeks. Two technological repeats present a composite pool including 5 mice per group. One representative result from three-independent experiments is shown here.

f) Relative plasma FGF21 (top) and liver FGF21 (bottom), normalized by R250 signaling or ACTIN respectively, is shown here as a bar graph. Data are represented as mean ± SEM (*n*=5 per group, two technological repeats present a composite pool). ns, $p > 0.05$; *, $p < 0.05$; **, $p < 0.01$; p values were determined by two-way ANOVA followed Tukey's multiple comparisons test. Source data for this figure are provided as a Source data file.

Supplementary Table 1. Primers for Quantitative PCR analysis

Gene Name	**Forward primer**	**Reverse primer**	**Organism**
Acc	TGACAGACTGATCGCAGAGAAAG	TGGAGAGCCCCACACACA	mouse
Acl	GCCAGCGGGAGCACATC	CTTTGCAGGTGCCACTTCATC	mouse
Acox1	TTTGTTGTCCCTATCCGTGAGA	CCGATATCCCCAACAGTGATG	mouse
Acox2	CATCCAACGTGACCCAGTGTT	AAATGCGTTCAGGACCGTCTT	mouse
Acsl1	CGCACCCTTCCAACCAACA	CGCTATTTCCACTGACTGCAT	mouse
ApoB	CGTGGGCTCCAGCATTCTA	TCACCAGTCATTTCTGCCTTTG	mouse
ApoE	GCTGGGTGCAGACGCTTT	TGCCGTCAGTTCTTGTGTGACT	mouse
Atgl	ATGTTCCCGAGGGAGACCAA	GAGGCTCCGTAGATGTGAGTG	mouse
Cebpa	GCGGGAACGCAACAACATC	GTCACTGGTCAACTCCAGCAC	mouse
Cel	CGCCTGGAGGTTCTATTTCTTG	TCCACGAAACCGCCTTCTG	mouse
Cpt1a	CGGAGACGACGCTTTCGAC	CGTAGTTGGAAGTACACCAGGA	mouse
Creb	GTCCCAGGCTCTCTATCATCTC	ATAGGCATCAAGACGGCAGAA	mouse
Crtc2	CACCAGAACTTGACCCACTGT	CACAGGGGTCACTCAGCATAG	mouse
Fasn	GCTGCGGAAACTTCAGGAAAT	AGAGACGTGTCACTCCTGGACTT	mouse
G6pc	TCTGTCCCGGATCTACCTTG	GTAGAATCCAAGCGCGAAAC	mouse
Glut4	ACACTGGTCCTAGCTGTATTCT	CCAGCCACGTTGCATTGTA	mouse
Hmgcl	CCGGCATCAACTACCCAGTC	GCGCTGGAAACTCTCCTCTAT	mouse
Hmgcs	GCCGTGAACTGGGTCGAA	GCATATATAGCAATGTCTCCTGCAA	mouse
Hsl	TGGCACACCATTTTGACCTG	TTGCGGTTAGAAGCCACATAG	mouse
Insig1	TCACAGTGACTGAGCTTCAGCA	TCATCTTCATCACACCCAGGAC	mouse
Insig2b	CCGGGCAGAGCTCAGGAT	GAAGCAGACCAATGTTTCAATGG	mouse
L32	TCTGGTGAAGCCCAAGATCG	CTCTGGGTTTCCGCCAGTT	mouse
Ldlr	GAGGAACTGGCGGCTGAA	GTGCTGGATGGGGAGGTCT	mouse
Lipg	ATGCGAAACACGGTTTTCCTG	GGACGCAAGGTTGTGATACTTC	mouse
Lpl	TTGCCCTAAGGACCCCTGAA	TTGAAGTGGCAGTTAGACACAG	mouse
Lxra	ACAGAGCTTCGTCCACAAAAG	GCGTGCTCCCTTGATGACA	mouse
Mgll	AGGCGAACTCCACAGAATGTT	ACAAAAGAGGTACTGTCCGTCT	mouse
Mttp	ATACAAGCTCACGTACTCCACT	TCTCTGTTGACCCGCATTTTC	mouse
Pck1	GTGCTGGAGTGGATGTTCGG	CTGGCTGATTCTCTGTTTCAGG	mouse
Pcsk9	ACCCTCATAGGCCTGGAGTT	CTGTGATGACCTCTGGAGCA	mouse
Pgc1a	TGCAAGACCGTGGTGCCACC	TCCTCGGCTGAGCCCTGAGG	mouse
Pgc1b	AGCTGCTTCTGTCTGTGAGTTTCC	AAGGGGCGATGGGTGACGGA	mouse
Ppara	TCTGTGGGCTCACTGTTCT	AGGGCTCATCCTGTCTTTG	mouse
Pparg	GGCTGAGGAGAAGTCACACTCTG	AAATCTTGTCTGTCACACAGTCCTG	mouse
Retn	TCTCCTCCAGAGGGAAGTTGG	TTTCTTCACGAATGTCCCACG	mouse
Rxra	ATGGACACCAAACATTTCCTGC	CCAGTGGAGAGCCGATTCC	mouse
Scap	ATTTGCTCACCGTGGAGATGTT	GAAGTCATCCAGGCCACTACTAATG	mouse

Scd1	CCGGAGACCCCTTAGATCGA	TAGCCTGTAAAAGATTTCTGCAAACC	mouse
Srebf-1a	GGCCGAGATGTGCGAACT	TTGTTGATGAGCTGGAGCATGT	mouse
Srebf-1c	GCGGAGCCATGGATTGCAC	CTCTTCCTTGATACCAGGCCC	mouse
Srebf-2	GCGTTCTGGAGACCATGGA	ACAAAGTTGCTCTGAAAACAAATCA	mouse
Ucp1	AGGCTTCCAGTACCATTAGGT	CTGAGTGAGGCAAAGCTGATTT	mouse
Ucp2	CAGCGCCAGATGAGCTTTG	GGAAGCGGACCTTTACCACA	mouse
Ucp3	CTGCACCGCCAGATGAGTTT	ATCATGGCTTGAAATCGGACC	mouse
Tnfaip2	AGGAGGAGTCTGCGAAGAAGA	GGCAGTGGACCATCTAACTCG	mouse
Pdk1	TCACAGATTTTGGAACAGCAA	TGAGCAGCTCTGGAGAAACA	mouse
C2cd4a	CTCTTGCGGGACCGAGATG	GGTCTGGAGTGAGCACGTT	mouse
Gcg	TGAATGAAGACAAACGCCACT	CCACTGCACAAAATCTTGGGC	mouse

Supplementary Table 2. Primers for CHIP-QPCR analysis

**Primer name**	**Detect site**	**Organism**	**Usage**	**Sequence**
CRE-*G6pc*-F	CRE	Mouse	CHIP	GGAGGGCAGCCTCTAGCACTGTCAA
CRE-*G6pc*-R	CRE	Mouse	CHIP	TCAGTCTGTAGGTCAATCCAGCCCT
CRE-*Pck1*-F	CRE	Mouse	CHIP	TCTCCCTGGAGTTTATTGTG
CRE-*Pck1*-R	CRE	Mouse	CHIP	TACTATATAGAAGGGAGGACAGC
CRE-*Pgc1α*-F	CRE	Mouse	CHIP	GGTTTAGAGTTGGTGGCATT
CRE-*Pgc1α* -R	CRE	Mouse	CHIP	CACCTGTCTTACTACAGTCCC
CRE-*Lxrα*-F	Half CRE	Mouse	CHIP	ATGGGAAGACAAACCACTAAA
CRE-*Lxrα*-R	Half CRE	Mouse	CHIP	AACGCAGGGAGGGCTAT
LXRE-*Srebp1*-F	LXRE	Mouse	CHIP	CTTGCTGCTGCCATTCG
LXRE-*Srebp1*-F	LXRE	Mouse	CHIP	GGGTTTCTCCCGGTGCT

Supplementary Table 3. The antibody, plasmids and other materials used in this work

Type	Name	Provider	Note
Antibodies	Rabbit anti-CREB	Cell Signal Technology (CST, #9197)	1:1000
Antibodies	Rabbit anti-CRTC2 pAb (454-607)	Merck (ST1099)	1:8000
Antibodies	Rabbit anti-HISTON H3	Abcam (ab1791)	1:2000
Antibodies	Rabbit anti-HISTON H3K27ac	Abcam (ab4729)	1:2000
Antibodies	Rabbit anti-CBP (D6C5)	Cell Signal Technology (CST, #7389)	1:2000
Antibodies	Rabbit anti-SREBP1 (2A4)	Santa Cruze (sc-13551)	1:2000
Antibodies	mouse anti-SREBP2	BD Biosciences (557037)	1:2000
Antibodies	Rabbit anti-AMPKα (D5A2)	Cell Signal Technology (CST, #5831S)	1:2000
Antibodies	Rabbit anti-P-Thr172-AMPKα (40H9)	Cell Signal Technology (CST, #2535S)	1:2000
Antibodies	Rabbit anti-HSP90(4F10)	Santa Cruze (sc-69703)	1:2000
Antibodies	Rabbit anti-LXRα	Abcam (ab190727)	1:2000
Antibodies	Rabbit anti-RXRα	Cell Signal Technology (CST, #3085)	1:2000
Antibodies	Rabbit anti-PPARα	CAYMAN Chemical Company (041071)	1:2000
Antibodies	Mouse anti-GAPDH	AGOMA (AGM90111)	1:2000
Antibodies	Rabbit anti-MYC	Cell Signal Technology (CST, #18583)	1:2000
Antibodies	Rabbit anti-HA	Cell Signal Technology (CST, #3724)	1:2000
Antibodies	Rabbit anti-PGC1α	Merck (ABE868-25UG)	1:2000
Antibodies	Rabbit anti-PEPCK (H-300)	Santa Cruze (sc-32879)	1:2000
Antibodies	Mouse anti-HIS (2A8)	Abmart (M20001)	1:2000
Antibodies	Rabbit anti-GST (4C10)	Covance (MMS-112R)	1:2000
Antibodies	Mouse anti- α -TUBULIN	Abmart (T40103)	1:5000
Antibodies	Mouse anti-ACTIN	Abmart (T40104)	1:5000
Antibodies	Mouse anti-FLAG (M2)-HRP	SIGMA (AB592)	1:5000
Antibodies	Rabbit anti-AKT	Cell Signal Technology (CST # 9272)	1:2000
Antibodies	Rabbit anti-Phospho-AKT-(Ser473)	Cell Signal Technology (CST #9271)	1:2000
Plasmid	811-*Creb*-BD	human CREB fused with GAL4 BD domain (binding DNA domain)	
Plasmid	804-*Crtc2*(S171A)-AD	human CRTC2 fused with VP16 AD domain (active domain).	
Plasmid	PM-BD-*Cbp*-KIX	KIX domain of CBP fused with BD (binding DNA domain)	
Plasmid	VP-AD-*Creb*-KID	KID domain of CREB fused VP16 AD domain (active domain).	
Plasmid	pGX5X-GST-*Creb*	human CREB fused N' terminal GST tag	
Plasmid	pET28C-HIS-*Crtc2*(S171A)	human CRTC2 fused N' terminal HIS tag	
Plasmid	HA-*P300*	mouse P300 fused N' terminal HA tag	

Plasmid	*GFP-Crtc1*	mouse CRTC1 fused N' terminal GFP	
Plasmid	*GFP-Crtc3*	mouse CRTC1 fused N' terminal GFP	
Plasmid	*MYC-Creb*	human CREB fused N' terminal MYC tag	
Plasmid	*FLAG-Crtc2*	mouse CRTC2 fused N' terminal FLAG tag	
Plasmid	*HA-Sec31A*	mouse SEC31A fused N' terminal HA tag	
AD-Virus	AD-*CRE-LUC*	AD vrius contain CRE driven luciferase reporter	
AD-Virus	AD-*G6p-LUC*	AD vrius contain *G6p* promoter driven luciferase reporter	
AD-Virus	AD-*β-Gal*	AD vrius contain RSV promoter driven β-gal reportet	
AD-Virus	AD-*HA-Crtc2*	AD virus contains mouse CRTC2 fused with N’ terminal HA tag	
Lenti-Virus	LV-*Crtc2-FLAG-GFP*	Lenti-virus contain CRTC2 fused C’ FLAG tag, and co-expressed GFP	
Peptides	glucagon, (1-29)	GL Biochem (Shanghai) Ltd.（GLS52256）	
inhibitor, chemical	Metformin	SIGMA (PHR1084-500MG)	
inhibitor, chemical	Tristatin, TSA	Selleck (S1045)	
Critical commercial assays	GO assay,	SIGMA (GAGO20)	
Critical commercial assays	LANCE Ultra cAMP Detection Kit	Perkin Elmer (TRF0262)	
Critical commercial assays	SYBR Real-time PCR mix	TAKARA (DRR041A)	
Experimental animal Models	*db/db* mice, C57BL/6J	The Jackson Laboratory	
Experimental animal Models	*Crtc2*-KO mice, C57BL/6J	Gift of Wangyiguo Laboratory	
Experimental animal Models	High fat diet induced mice	Research Diet, 60% fat， D12492	

Oligo-nucleotides	qPCR primer as list		
Software	Prism8.0		
Software	Origin 9.0		
Software	Quantity One		
Equipment	ABI7900 Real-time PCR system	ABI	
Equipment	CLAMS open-circuit indirect calorimetry	Columbus Instruments	
Equipment	Accu-Chek® Inform II system;	Roche, Glucometer and strips	
Equipment	Nano Drop 2000	Thermo Fishe	
Equipment	The minispec Live Mice Analyzer	Nuclear magnetic resonance, NMR, Bruker, LF50	
Equipment	Imaging System	Xenogen, IVIS-100	
Equipment	EnVision Multimode Plate Reader	Perkin/Elmer, EnVision 2105	
Equipment	BIACORE 100	GE Healthcare	
Equipment	MicroCal iTC200 calorimeter	Freiburg, Germany	
Equipment	Liquid chromatography-mass spectrometry/mass spectrometry (LC-MS/MS) system	Thermo Fisher	
Equipment	WinNonLin professional version 4.1	Pharsight Corp., Mountain View, CA	
Equipment	HNMR	Varian-MERCURY Plus-400 or BRUKER BIOSPIN AG AVANCE III 500	
Equipment	CNMR	BRUKER BIOSPIN AG AVANCE III 500	
Equipment	HPLC	Agilent 1260	

Supplementary Table 4. The structure of CREB/CRTC2 inhibitors and their inhibitory activity.

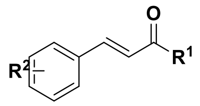

Compd.	R^1	R^2	IC_{50} (μM)
**A32**	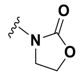	**2-CF_3**	9.95
**A35**	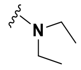	**2-CF_3**	53.3
**A37**	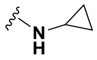	**2-CF_3**	> 100
**A40**	**-OH**	**2-CF_3**	> 100
**A43**	**-OMe**	**2-CF_3**	81.1
**A47**	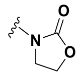	**3-CF_3**	13.8
**A50**	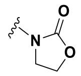	**4-CF_3**	18.7
**A53**	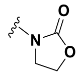	**2-OMe**	3.1
**A54**	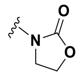	**3-OMe**	1.5
**A56**	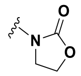	**4-OMe**	39.7
**A57**	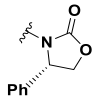	**3-Ph**	0.74
**A58**	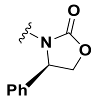	**3-Ph**	73.5
**A1101**	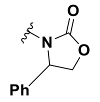	**3-Ph**	3.56
**APC**			27.4
