# Supplementary material for: A propolis-derived small molecule ameliorates metabolic syndrome in obese mice by targeting the CREB/CRTC2 transcriptional complex
Source: Nat Commun. 2022 Jan 11;13:246. doi: 10.1038/s41467-021-27533-9 (PMC8752738; doi:10.1038/s41467-021-27533-9)
Supplement: Supplementary file 2 — Reporting Summary [file 41467_2021_27533_MOESM2_ESM.pdf]

nature research

Corresponding author(s): Lihong Hu, Hong Liu & Yi Liu

Last updated by author(s): 6/22/21

Reporting Summary

Nature Research wishes to improve the reproducibility of the work that we publish. This form provides structure for consistency and transparency in reporting. For further information on Nature Research policies, see our Editorial Policies and the Editorial Policy Checklist.

Statistics

For all statistical analyses, confirm that the following items are present in the figure legend, table legend, main text, or Methods section.

n/a	Confirmed	
☐	☒	The exact sample size (n) for each experimental group/condition, given as a discrete number and unit of measurement
☐	☒	A statement on whether measurements were taken from distinct samples or whether the same sample was measured repeatedly
☐	☒	The statistical test(s) used AND whether they are one- or two-sided<br>*Only common tests should be described solely by name; describe more complex techniques in the Methods section.*
☐	☒	A description of all covariates tested
☐	☒	A description of any assumptions or corrections, such as tests of normality and adjustment for multiple comparisons
☐	☒	A full description of the statistical parameters including central tendency (e.g. means) or other basic estimates (e.g. regression coefficient) AND variation (e.g. standard deviation) or associated estimates of uncertainty (e.g. confidence intervals)
☐	☒	For null hypothesis testing, the test statistic (e.g. F, t, r) with confidence intervals, effect sizes, degrees of freedom and P value noted<br>*Give P values as exact values whenever suitable.*
☒	☐	For Bayesian analysis, information on the choice of priors and Markov chain Monte Carlo settings
☒	☐	For hierarchical and complex designs, identification of the appropriate level for tests and full reporting of outcomes
☒	☐	Estimates of effect sizes (e.g. Cohen's d, Pearson's r), indicating how they were calculated

Our web collection on statistics for biologists contains articles on many of the points above.

Software and code

Policy information about availability of computer code

Data collection	Applied Biosystems 7900HT Fast Real-Time PCR System (ABI, SDS software 2.0);<br>Quantity One® 1-D (Bio-Rad);<br>Accu-Chek® Inform II system (Roche, Glucometer and strips);<br>Nano Drop 2000 (Thermo Fisher);<br>The minispec Live Mice Analyzer (Nuclear magnetic resonance, NMR, Bruker, LF50);<br>Imaging System (Xenogen, IVIS-100);<br>EnVision Multimode Plate Reader (Perkin/Elmer, EnVision 2105);<br>BIACORE 100 (GE Healthcare);<br>MicroCal iTC200 calorimeter (Freiburg, Germany);<br>Comprehensive Lab Animal Monitoring System (CLAMS-16);<br>Liquid chromatography-mass spectrometry/mass spectrometry (LC-MS/MS) system (Thermo Fisher);<br>WinNonLin professional version 4.1 (Pharsight Corp., Mountain View, CA);<br>HNMR （Varian-MERCURY Plus-400 or BRUKER BIOSPIN AG AVANCE III 500);<br>CNMR (BRUKER BIOSPIN AG AVANCE III 500);<br>HPLC (Agilent 1260).

Data analysis	Origin9.0 (Origin Lab);<br>Living Image Software (Xenogen, IVIS-100);<br>Quantity One® 1-D (Bio-Rad);<br>Graphpad8.0 (Prism);

For manuscripts utilizing custom algorithms or software that are central to the research but not yet described in published literature, software must be made available to editors and reviewers. We strongly encourage code deposition in a community repository (e.g. GitHub). See the Nature Research guidelines for submitting code & software for further information.

Data

Policy information about availability of data

All manuscripts must include a data availability statement. This statement should provide the following information, where applicable:
- Accession codes, unique identifiers, or web links for publicly available datasets
- A list of figures that have associated raw data
- A description of any restrictions on data availability

The authors declared that the data supporting the findings of this study were available within the paper and supplementary information files. And a list of figures were associated with raw data.

Field-specific reporting

Please select the one below that is the best fit for your research. If you are not sure, read the appropriate sections before making your selection.

[x] Life sciences [ ] Behavioural & social sciences [ ] Ecological, evolutionary & environmental sciences

For a reference copy of the document with all sections, see nature.com/documents/nr-reporting-summary-flat.pdf

Life sciences study design

All studies must disclose on these points even when the disclosure is negative.

Sample size	Given that the animal supplier has identified leptin-receptor knockout and overweight phenotypes of db/db mice, we used 5-7 db/db mice per group in vivo, such as Fig 1a. For DIO mice, there were 10-12 mice per group, for example Fig. 4X. This sample size was generally accepted in the field of metabolism research, for example db/db (n=3) in article Figure 1A and 1B (DOI 10.1016/j.cmet.2009.01.007) of CELL Metabolism.
Data exclusions	When mice died or were missing during experiment, we excluded the data of these mice. In Fig. 4a, one mouse i.p. APC 10mg/kg was missing. And in Fig. 4b, a mouse i.p. vehicle died. No exclusion criteria were pre-established.
Replication	At least three successful and independent experiments were included in the analysis. The independent cellular experiments were repeated continuously, generally one time each week.
Randomization	In this study we used primary hepatocytes, HEK293T cell line and the purified protein in vitro assay. These homogenous biological materials were allocated into the indicated groups according to the experiment design. The results of assays were related with designed treatment. Also, the covariates in these assays were determined by ANOVA.
Blinding	In vivo assay, the investigators were blinded to data collection and/or analysis, which were performed by another researcher. In compound screening assay, the investigators were blinded for structure and name information of test compounds.

Reporting for specific materials, systems and methods

We require information from authors about some types of materials, experimental systems and methods used in many studies. Here, indicate whether each material, system or method listed is relevant to your study. If you are not sure if a list item applies to your research, read the appropriate section before selecting a response.

Materials & experimental systems

n/a	Involved in the study	
[ ]	[x]	Antibodies
[ ]	[x]	Eukaryotic cell lines
[x]	[ ]	Palaeontology and archaeology
[ ]	[x]	Animals and other organisms
[x]	[ ]	Human research participants
[x]	[ ]	Clinical data
[x]	[ ]	Dual use research of concern

Methods

n/a	Involved in the study	
[x]	[ ]	ChIP-seq
[x]	[ ]	Flow cytometry
[x]	[ ]	MRI-based neuroimaging

Antibodies

Antibodies used	*Describe all antibodies used in the study; as applicable, provide supplier name, catalog number, clone name, and lot number.*
Validation	The authors declared the validation of species and applications for these anti-bodies used in this manuscript, and the validity of statements on the manufacturer's website.

Eukaryotic cell lines

Policy information about cell lines

Cell line source(s)	The HEK293T cell line was purchased from the National Collection of Authenticated Cell Cultures (CAS)（WWW..
Authentication	This authenticated cell line was purchased from National Collection of Authenticated Cell Cultures, Shanghai, China (NICR).
Mycoplasma contamination	The mycoplasma contamination test of this cell line was negative.
Commonly misidentified lines (See ICLAC register)	There were no commonly misidentified lines used in this manuscript.

Animals and other organisms

Policy information about studies involving animals; ARRIVE guidelines recommended for reporting animal research

Laboratory animals	This work used laboratory animals including db/db (BKL6), C57/BL6 and ICR/JCL mice. The three weeks wild male C57/BL6 mice were fed a high fat diet (HF) for 13 weeks to setup the diet induced obesity (DIO) animal models. And the seven weeks male db/db (BKL6) or ICR/JCL mice were applied for in vivo assay.
Wild animals	The authors declared that no wild animals were used in this study.
Field-collected samples	The authors declared that no field-collected samples were applied in this study.
Ethics oversight	All animal experiments were performed according to procedures approved by the Shanghai Institutes for Biological Sciences,CAS (SIBS. CAS) Animal Care and Use Committee

Note that full information on the approval of the study protocol must also be provided in the manuscript.
